# Pathological features of post-stroke pain: a comprehensive analysis for subtypes

**DOI:** 10.1093/braincomms/fcaf128

**Published:** 2025-04-30

**Authors:** Yuki Igawa, Michihiro Osumi, Yusaku Takamura, Hidekazu Uchisawa, Shinya Iki, Takeshi Fuchigami, Shinji Uragami, Yuki Nishi, Nobuhiko Mori, Koichi Hosomi, Shu Morioka

**Affiliations:** Graduate School of Health Science, Kio University, Kitakatsuragi-gun, Nara 635-0832, Japan; Department of Rehabilitation Medicine, Nishiyamato Rehabilitation Hospital, Kitakatsuragi-gun, Nara 639-0218, Japan; Graduate School of Health Science, Kio University, Kitakatsuragi-gun, Nara 635-0832, Japan; Neurorehabilitation Research Center, Kio University, Kitakatsuragi-gun, Nara 635-0832, Japan; Neurorehabilitation Research Center, Kio University, Kitakatsuragi-gun, Nara 635-0832, Japan; Graduate School of Health Science, Kio University, Kitakatsuragi-gun, Nara 635-0832, Japan; Department of Rehabilitation Medicine, Nishiyamato Rehabilitation Hospital, Kitakatsuragi-gun, Nara 639-0218, Japan; Department of Rehabilitation Medicine, Kawaguchi Neurosurgery Rehabilitation Clinic, Hirakata-shi, Osaka 573-0086, Japan; Neurorehabilitation Research Center, Kio University, Kitakatsuragi-gun, Nara 635-0832, Japan; Department of Rehabilitation, Kishiwada Rehabilitation Hospital, Kishiwada-shi, Osaka 596-0827, Japan; Graduate School of Health Science, Kio University, Kitakatsuragi-gun, Nara 635-0832, Japan; Department of Rehabilitation, Hoshigaoka Medical Center, Hirakata-shi, Osaka 573-0013, Japan; Neurorehabilitation Research Center, Kio University, Kitakatsuragi-gun, Nara 635-0832, Japan; Institute of Biomedical Sciences (Health Sciences), Nagasaki University, Nagasaki-shi, Nagasaki 852-8520, Japan; Department of Neurosurgery, Osaka University Graduate School of Medicine, Suita, Osaka 565-0871, Japan; Department of Neurosurgery, Osaka University Graduate School of Medicine, Suita, Osaka 565-0871, Japan; Graduate School of Health Science, Kio University, Kitakatsuragi-gun, Nara 635-0832, Japan; Neurorehabilitation Research Center, Kio University, Kitakatsuragi-gun, Nara 635-0832, Japan

**Keywords:** PSP, CPSP, pathological feature, comprehensive assessment

## Abstract

Post-stroke pain is heterogeneous and includes both nociceptive and neuropathic pain. These subtypes can be comprehensively assessed using several clinical tools, such as pain-related questionnaires, quantitative somatosensory tests and brain imaging. In the present study, we conducted a comprehensive assessment of patients with central post-stroke pain and non-central post-stroke pain and analysed their clinical features. We also performed a detailed analysis of the relationships between brain lesion areas or structural disconnection of the white matter and somatosensory dysfunctions. In this multicentre cross-sectional study, 70 patients were divided into 24 with central post-stroke pain, 26 with non-central post-stroke pain and 20 with no-pain groups. Multiple logistic regression analysis was used to summarize the relationships between each pathological feature (for the central post-stroke pain and non-central post-stroke pain groups) and pain-related factors or the results of quantitative somatosensory tests. Relationships between somatosensory dysfunctions and brain lesion areas were analysed using voxel-based lesion–symptom mapping and voxel-based disconnection–symptom mapping. All pathology feature models indicated that central post-stroke pain was associated with cold hypoesthesia at 8°C (β = 2.98, odds ratio = 19.6, 95% confidence interval = 2.7–141.8), cold hyperalgesia at 8°C (β = 2.61, odds ratio = 13.6, 95% confidence interval = 1.13–163.12) and higher Neuropathic Pain Symptom Inventory scores (for spontaneous and evoked pain items only; β = 0.17, odds ratio = 1.19, 95%, confidence interval = 1.07–1.32), whereas non-central post-stroke pain was associated with joint pain (β = 5.01, odds ratio = 149.854, 95%, confidence interval = 19.93–1126.52) and lower Neuropathic Pain Symptom Inventory scores (β = −0.17, odds ratio = 0.8, 95%, confidence interval = 0.75–0.94). In the voxel-based lesion–symptom mapping, the extracted lesion areas indicated mainly voxels significantly associated with cold hyperalgesia, allodynia at 8°C and 22°C and heat hypoesthesia at 45°C. These extracted areas were mainly in the putamen, insular cortex, hippocampus, Rolandic operculum, retrolenticular part of internal and external capsules and sagittal stratum. In voxel-based disconnection–symptom mapping, the extracted disconnection maps were significantly associated with cold hyperalgesia at 8°C, and heat hypoesthesia at 37°C and 45°C. These structural disconnection patterns were mainly in the cingulum frontal parahippocampal tract, the reticulospinal tract and the superior longitudinal fasciculus with a widespread interhemispheric disconnection of the corpus callosum. These findings serve as important indicators to facilitate decision-making and optimize precision treatments through data dimensionality reduction when diagnosing post-stroke pain using clinical assessments, such as bedside quantitative sensory testing, pain-related factors, pain questionnaires and brain imaging.

## Teaser

Igawa *et al*. reported that central post-stroke pain was characterized by cold hypoesthesia, cold hyperalgesia and higher pain scores, which were correlated to brain lesions affecting cortical or subcortical networks. In contrast, other pain types were associated with joint pain and lower pain scores.

## Graphical Abstract

**Graphical Abstract:**
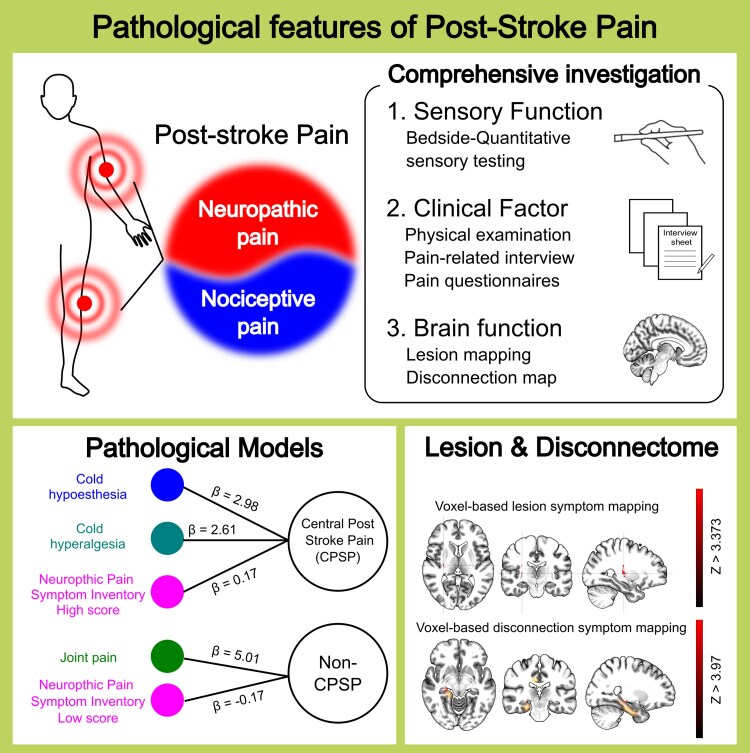

## Introduction

Stroke patients experience not only motor or sensory dysfunctions and cognitive dysfunction but also pain known as ‘post-stroke pain’ (PSP).^1^ PSP can occur subacutely or several years after stroke,^1-4^ and its incidence varies from approximately 10.6% to 55%.^1-8^ PSP can be broadly divided into nociceptive pain and neuropathic pain^1,2^ and can have extremely negative influences on patients’ treatments and daily life.^7,9,10^ Nociceptive pain in PSP comprises post-stroke shoulder pain, musculoskeletal pain and spasticity. Neuropathic pain in PSP is also known as central post-stroke pain (CPSP), which presents as spontaneous pain and evoked pain.^1-3^ PSP is therefore generally divided into non-CPSP and CPSP.

Previous studies have investigated the pathologies of non-CPSP and CPSP. For example, in CPSP, a study of macaque monkeys revealed that specific pain and temperature hypersensitivity appear after thalamic lesions.^11^ CPSP has also been reported to occur with lesions to the pain pathway.^12-15^ Further clinical studies have demonstrated that lesions of both the thalamus and regions other than the thalamus can cause CPSP, leading to allodynia or hyperalgesia, hypersensitivity and hypoesthesia.^16-25^ In the case of non-CPSP (e.g. post-stroke shoulder pain or musculoskeletal pain), pain is thought to derive from tissue injury.^26-28^ For example, reduced motor function caused by haemiplegia can cause tissue injury and thus shoulder joint pain.^28^ CPSP pathology is therefore strongly associated with central lesions, whereas non-CPSP pathology is associated with peripheral tissue injury. However, it is difficult to capture all of the pathological features of each patient in clinical practice because two or more pathological mechanisms are often present in a single PSP patient.^29^ A comprehensive assessment—including somatosensory assessments (e.g. spontaneous pain and evoked pain), physical function evaluations and diagnostic brain imaging—are important for understanding the pathological features of each patient.^30^ Nonetheless, most previous studies have not comprehensively assessed PSP patients; instead, they have focused on a single subdivided pathology of PSP.^12-29^ That is, some studies have focused on CPSP only, whereas other studies have focused on non-CPSP only. However, because the pathological features of PSP overlap, namely, it is heterogeneous, clinical studies of PSP that include both non-CPSP and no-pain groups are needed to dissect what is specific or coexisting and systematically model this disorder. The heterogeneity underlying these symptoms should be investigated in detail to better identify the distinguishing clinical indicators for CPSP and non-CPSP. In addition, combined quantitative sensory testing (QST),^31,32^ voxel-based lesion–symptom mapping (VLSM),^33^ and voxel-based disconnection–symptom mapping (VDSM)^34^ are more useful for investigating the relevant brain lesion based on symptoms of CPSP or non-CPSP in detail. We hypothesized that analysing the relationships between somatosensory phenotypes and brain lesion areas and pathways will help to reveal the detailed pathology of CPSP, whereas the non-CPSP pathology will make clear that factors are unrelated.

In the present study, we conducted a comprehensive assessment of patients in non-CPSP, CPSP and no-pain groups, analysing their clinical features. In addition, we analysed the relationships between brain lesion areas, structural disconnection (SDC) of the white matter and somatosensory dysfunctions in non-CPSP and CPSP patients to understand the underlying pathology.

## Materials and methods

### Participants

This cross-sectional study was performed on stroke patients in subacute or chronic phases (Table 1). Patients participated in this study as outpatients or while hospitalized in one of four affiliated institutions. Participants with ischaemic or haemorrhagic stroke confirmed by imaging (CT or MRI) were enrolled in this study. In addition, three patients with a history of a previous stroke were also included in this study. They had an injury on one side only, as seen by the small lacunar infarctions, which had no symptoms before the current onset (the contralateral areas were unremarkable). Exclusion criteria were an acute stroke (defined as within 1 month from onset), a diagnosis of transitory cerebral ischaemic attack or subarachnoid haemorrhage, communication problems (e.g. severe aphasia or dysarthria), severe dementia, somnolence, a lack of understanding of questionnaires, the presence of nociceptive and neuropathic pain in other diseases, or clear sensory disturbances and/or motor impairments caused by bilateral stroke lesions. Inclusion criteria were patients aged >18 years; we recruited patients with subacute and chronic stroke between 1 April 2020 and 31 March 2024. In the 50 patients with pain, the condition was that pain occurred after stroke. The recruited patient participants had not reported pain previously. We confirmed that if a patient had previously experienced pain, the pain caused after the stroke occurred in a different location to the previous areas (e.g. if a patient had pain from a compression fracture previously, the pain areas, such as the forearm and finger, that occurred after the stroke were not associated). We further divided the participants into three groups: (i) patients with CPSP, (ii) patients with non-CPSP and (iii) patients with no pain.

**Table 1:** Demographics of patients included in the statistical analysis

				Significance tests		
Demographics and characteristics	CPSP (*N* = 24)	Non-CPSP (*N* = 26)	No-pain (*N* = 20)	CPSP versus non-CPSP	CPSP versus no-pain	Non-CPSP versus no-pain
Age, mean (SD)	63.8 (±9.77)	70.6 (±11.15)	75.9 (±8.58)	<0.05*	<0.05*	0.08
Sex, n (%)						
Male	75	53.8	65	0.44	1	1
Days from stroke, median (range)	133.5 (77.5–199.25)	61.5 (44–89.5)	72 (36.75–91.5)	<0.05*	<0.05*	0.86
Days of pain duration, median (range)	49 (14–162.75)	37 (15.25–52.5)		0.26		
Stroke type, *n* (%)						
Haemorrhage	62.5	34.6	40.0	0.26	0.45	0.76
Cerebral infarction	37.5	65.4	60.0			
Haemiplegia side, n (%)						
Right	41.7	26.9	65.0	0.37	0.28	<0.05*
Left	58.3	73.1	35.0			
Pain limb side, n (%)						
Right	41.7	26.9		0.37		
Left	58.3	73.1				
Pain sites						
Pain segmented the upper or lower limb, n (%)	18 (75)	4 (15)		<0.05*		
Localized pain, n (%)	6 (25)	22 (84.6)		<0.05*		
Pain medication✝, n (%)						
Total	7 (41.2)	4 (20)		0.13		
Anti-inflammatory	6	5				
Anti-convulsant	1					
Opioid		1				
Pregabalin	4					
Mean pain intensity	4.95 ± 2.63	4.5 ± 2.0		0.26		

Differences between groups: the multiple comparisons test was used for age, stroke time, and pain time. Fisher's exact test was used with sex, stroke type, hemi side, pain side, pain sites, and medication as the variables. *P < 0.05. ✝Pain medications were dosing anti-inflammatory, anti-convulsant, pregabalin and opioid medication. CPSP, central post-stroke pain; non-CPSP, non-central post-stroke pain.

**Figure 1:**
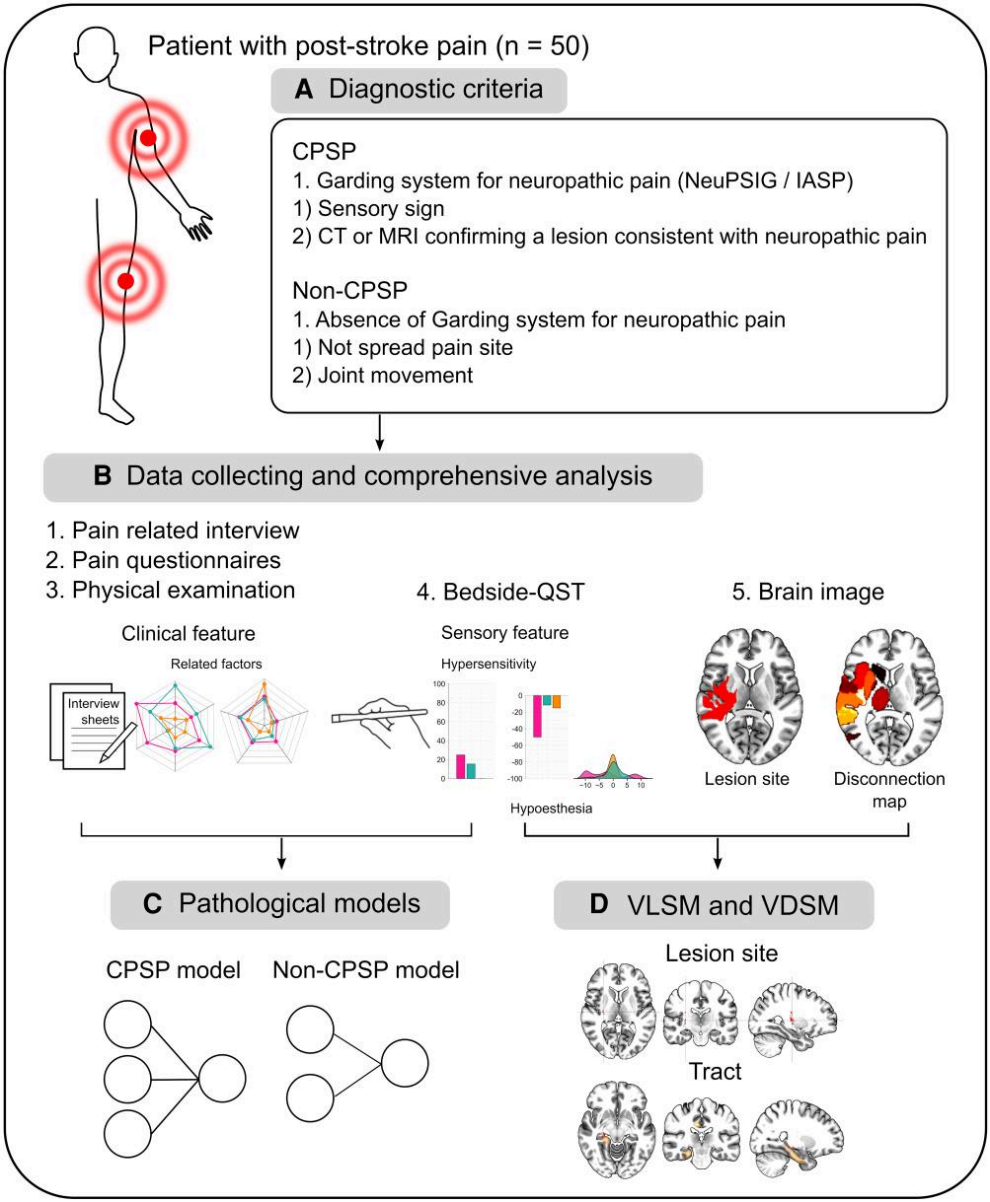
**Group criteria and framework of the study.** (**A**) The 50 patients with PSP were classified into 2 groups. The CPSP group was defined according to a grading system for neuropathic pain (NeuPSIG) and the diagnostic criteria identified by previous study.^2^ The non-CPSP group included participants who did not have neuropathic pain according to the grading system for diagnosis of neuropathic pain. (**B**) Data were analysed comprehensively for the three groups of patients who received assessments, including pain-related interviews, physical examinations, the bedside-QST and brain image. (**C**) To distinguish the CPSP and non-CPSP phenotypes, we performed multiple logistic regression analysis on these variables. (**D**) Brain image analysis was performed using VLSM and VDSM, which examine the relationships between brain lesions and pain/abnormal sensations in the bedside-QST. CPSP, central post-stroke pain; QST, quantitative sensory testing; VDSM, voxel-based disconnection–symptom mapping; VLSM, voxel-based lesion–symptom mapping.

Patients were classified into the CPSP group, which determines probable neuropathic pain if they had fulfilled one of the following two criteria from the International Association for Study of Pain Neuropathic Pain Special Interest Group (NeuPSIG) grading system for neuropathic pain (Fig. 1A): (i) sensory sign consistent with a lesion and (ii) CT or MRI confirming plausible lesion explaining neuropathic pain.^2,35,36^ If it was difficult to judge the CPSP by these criteria, we confirmed that the patient with CPSP had clearly abnormal sensations and spontaneous or evoked pain according to the supporting criteria.^2^ The pain distribution was necessarily conditioned to conform to the somatotopic representation of the body within the lesion. Patients with other types of pain were classified into the non-CPSP group if they had non-neuropathic pain (i.e. when joints of upper or lower limbs are moved, pain arises from contracted or shortened muscles because of increased muscle tension with spasticity after stroke, immobility and poststroke shoulder pain). This pain was localized to a specific site (Fig. 1), did not spread to multiple body regions and occurred immediately or within 2 months after stroke onset, persisting to the present time. Additionally, these patients exhibited no signs of neuropathic pain based on the NeuPSIG grading system. For patients with non-CPSP, pain duration was defined as temporary, arising from abnormal positions or repositioning limbs. If these patients had mixed pain (combining elements of both non-CPSP and CPSP), it was classified as nociceptive pain if it was consistent with the experienced pain when repositioning limbs in daily life and the pain experienced when moving joints in the assessments. Pain-free patients were classified into the no-pain group if they had no episodes of pain (including acute pain and translation to pain) during the clinical examination or later than 1 month after stroke onset. Moreover, in considering the possibility of initial symptoms of neuropathic pain, patients who had paresthesia were excluded. Thus, the no-pain group had only normal sensations or pure sensory disturbances without paresthesia, ensuring they did not develop pain later. All patients were assessed by pain examinations approximately 1 to 3 months after stroke onset.

This study was approved by the Ethics Committee of Kio University (reference number: R3-07). All procedures and potential risks were carefully explained, and written consent was obtained from all participants according to the Declaration of Helsinki.

### Comprehensive pain evaluation

All patients were assessed for pain in the admitted hospital or institution. The patients received assessments that included the bedside-QST, pain-related physical examination and pain questionnaire on activities of daily living (Fig. 1). We also investigated medical complications and the presence or absence of pain medications. Evaluations were performed at a time of day when each patient was less likely to be affected by medication. The questionnaire on pain, bedside-QST and pain-related interview and physical examination are detailed in the following text.

### Sensory phenotype for bedside quantitative sensory testing

Somatosensory functions of all patients were assessed using the bedside-QST, which was developed to assess somatosensory function at the bedside.^32^ The bedside-QST was performed at the area of the largest pain intensity (reference area was equally area with this location) in the affected upper or lower extremities, consistent with the brain lesion. Whereas, no-pain patients were performed at the affected dorsum of the hand in the upper extremities consistent with the brain lesion. The bedside-QST was conducted referencing the methodology reported in a previous study.^32^

### Thermal and pain sensations

Thermal and painful sensations were assessed using a Peltier Thermos controller device (VPE35-12-40S/VPE20-30S; VICS Inc, Tokyo, Japan) on the most painful area and the corresponding contralateral area. Patients were instructed to respond to the stimulus using the following procedure: (1) if patients felt the stimulation as painful, they noted the intensity of the pain using a numerical rating scale (NRS; 0 = no pain, 10 = worst imaginable pain); (2) if patients did not feel the stimulation as painful (i.e. they perceived it as only cold/warm), they noted the intensity of the thermal sensation using an NRS from 0 to 20 (10 = same intensity at the affected area as at the intact area, > 10 = stronger intensity of sensation at the affected area than at the contralateral area, < 10 = weaker intensity of sensation at the affected area than at the intact area). Temperatures were set at 37°C and 45°C to assess heat and heat-painful sensations, respectively, and at 22°C and 8°C to assess cold and cold-pain sensations, respectively. These stimuli were applied to the skin for 3 s.

### Touch sensation

The dynamic mechanical detection sensitivity evoked by a Q-tip stroke was assessed on the most painful area and the corresponding contralateral area (5-cm length, 1×). The participants with SDC were asked to describe whether they perceived the existence of the stimulus (yes/no). If they perceived the existence of the stimulus as a touch sensation, they were asked to note the intensity of the perception at the affected area compared with the control area using an NRS from 0 to 20 (10 = same intensity at the affected area as at the intact area, > 10 = stronger intensity of sensation at the affected area than at the contralateral area, < 10 = weaker intensity of sensation at the affected area than at the intact area).

### Mechanical pain intensity

To assess pinprick hyperalgesia and hypoesthesia, a 0.7-mm CMS hair (i.e. bedside pinprick) (Chicago Medical Supply, LLC, Chicago, IL, USA) was applied to the most painful area and the corresponding contralateral area. First, the patients had to indicate whether the stimulus was perceived as a blunt touch or as a pinprick. If the stimulus was perceived as a pinprick, the patients then had to rate the pain intensity using an NRS (0 = no pain, 10 = worst imaginable pain).

### Wind-up ratio

To assess temporal pain summation, the 0.7-mm CMS hair was applied over a series of 10 stimulations with a frequency of 1/s. Stimulation was applied to the most painful area and the corresponding contralateral area. The patients were instructed to rate the pain intensity induced by the first and last stimuli of the series using an NRS (0 = no pain, 10 = worst imaginable pain).

### Dynamic mechanical allodynia

To test for dynamic mechanical allodynia, patients were touched using a brush, a Q-tip and a cotton wisp on the most painful area and the corresponding contralateral area. These devices were used to draw a cross with two strokes (length: 5 cm) in each direction at a 90° angle. For each stimulus (brush/Q-tip/cotton wisp), the patients had to indicate (1) if the stimulus was perceived as painful (yes/no), and (2) the pain intensity on an NRS (0 = no pain, 10 = worst imaginable pain).

### Pressure pain sensitivity

A bedside algometer consisting of a 10-mL syringe sealed with a plug and felt (contact area 1 cm^2^) was placed above a muscle on the affected and intact sides. To assess the intensity of pressure pain, the syringe was compressed up to 4 mL and patients had to rate the pain intensity using an NRS (0 = no pain, 10 = worst imaginable pain). To assess the threshold of pressure pain, the syringe was then slowly compressed toward 1 mL/s, and patients were asked to respond when the pressure was perceived as painful [i.e. the pain threshold was determined by the amount of compressed air (in milliliter) in the syringe].

### Vibration detection threshold

The vibration detection threshold assessment was performed using a standard tuning fork (C-128 Hz/C-64 Hz, 8-point scale) placed over a bony prominence in the affected area and the corresponding contralateral area. Patients were asked to indicate when the vibration stimulus vanished (0 = no vibration stimulus perception, 8 = best possible vibration detection).

### Physical examination and pain of questionnaires

The following pain questionnaires were used for the CPSP and non-CPSP patients.

1. NRS: The Numerical Rating Scale consists of an 11-point from 0 to 10 scale (0; no pain, 10; worse imaginable pain) and recorded the mean pain intensity in activity daily life.
2. Short-form McGill Pain Questionnaires-2 (SF-MPQ-2): The SF-MPQ-2 consists of 18 sensory items and four affective items rated on a scale of 0 (none) to 10 (worst possible) and provides valuable information regarding individual pain characteristics.^37^ In particular, we were interested to attend to the sensory qualities of pain excluding the affective items of a total of SF-MPQ-2; 1. throbbing, 2. shooting, 3. stabbing, 4. sharp, 5. cramping, 6. gnawing, 7. hot-burning, 8. aching, 9. heavy, 10. tender, 11. splitting, 12. electric, 13. cold-freezing, 14. piercing, 15. light-touch, 16. itching, 17. tingling, 18. numbness.
3. Neuropathic Pain Symptoms Inventory (NPSI): The NPSI consists of 10 items of pain descriptors and 2 items to the duration of spontaneous ongoing and paroxysmal pain to assess the severity of neuropathic pain.^38^ This score consists of a total score ranging from 0 to 100, and each dimension's score ranging from 0 to 10 with higher scores indicating more intense symptoms. We analysed to focus on the spontaneous pain and the evoked pain associated with the mechanical and thermal allodynia.
4. Pain DETECT Questionnaire: The pain DETECT questionnaires to detect the neuropathic pain components consist of seven questions, the items of pain course pattern, and the presence or absence of radiating pain.^39^ We investigated the total scores of the pain DETECT questionnaires to compare between the CPSP and the Non-CPSP.
5. SF-PCS: The pain catastrophizing scale of long questionnaires to evaluate misinterpretation or overinterpretation of pain has been reported to validity and reliable. Recent studies have reported the development of short-form questionnaires within the PCS, which have been shown to be valid and reliable for assessment purposes.^40^ In the current study, we emphasized and analysed the total scores of the six items (items 4, 5, 6, 10, 11, and 13) short forms of PCS. The items categories consist of five response categories (0 = not all, 1 = to a slight degree, 2 = to a moderate degree, 3 = to a great degree and 4 = all the time).
6. Tampa Scale for Kinesiopahobia-11 (TSK-11): The TSK-11 is the questionnaire composed of 11 items (divided into two factors: somatic focus and activity avoidance), an abbreviated TSK 17-item instrument, designed to assess fear of movement or (re)injury; namely, it is used to assess the avoidance of physical activity.^41^ The total scores consist of the sum of all items of ranges from 1 to 4 points. A high total score suggests a strong potential for avoiding physical activity due to pain. We used the result of total scores in TSK-11.
7. Pain-related interview: The present study used a pain-related interview based on previous studies^42^ that referred to with or without joint pain triggered by joint movement, the mechanical and thermal allodynia, the limited range of motion and the subluxation of the shoulder joint. By assessing each item, this interview was used to assess whether the pain patients have these factors. This interview comprised five items (covering domains of sensory disturbance, triggered joint pain, restricted range of motion, subluxation of the shoulder joint and allodynia or hyperalgesia), answered using dichotomic variables (yes: 1, no: 0) (Supplementary Questionnaire). Finally, these items were calculated as proportions from binary values and quantified into percentages.
8. Physical examination: Motor function in upper and lower limbs was quantified by Fugl–Meyer Assessment (FMA), which higher values indicate more severe haemiplegia (score range of upper limb, 0–66, score range of lower limb, 0–34).^43^ Spasticity in the upper and lower limbs was assessed by the modified Ashworth scale, in which higher values indicate more severe spasticity.^44^ This score represents a 6-point score ranging from 0 to 4, with lower scores representing normal muscle tone, and higher scores representing spasticity or increased resistance to passive movement. The variables in the FMA were first calculated as a proportion (percentage) of the total score, whereas the modified Ashworth scale scores were calculated as a proportion to convert a numerical score of 1 or more in modified Ashworth scale items.

### Imaging and lesion analysis

Lesion analysis was performed by clinicians with abundant knowledge and experience in brain imaging diagnosis using CT or MRI data acquired in clinical practice. Typical imaging parameters were as follows; slice thickness = 5–10 mm, matrix = 512 × 512 for CT, 276–456 × 284–480 for MRI, field-of-view = 220–235.5, voxel size = 0.41–1.37 and number of slices = 20–49.

The latest scans for each patient were used for the present study. The mean duration between the stroke onset and MRI or CT was 63.7 ± 310.1 days (Supplementary Table 1). First, CT or MRI scans (T2-weighted or fluid-attenuated inversion recovery images) were used to identify the location(s) of lesion(s). If both scan modalities were available, MRI scans were selected. Each lesion was manually delineated on axial slices of the individual CT or MRI scans using MRIcron.^45^ If the patients had previous small lacunar infarctions, we delineated the lesion of the brain image, limiting it to the current onset. Normalization of the CT or MRI scans was then performed using the Clinical Toolbox^46^ in SPM12,^47^ which provides age-specific templates oriented in Montreal Neurological Institute space for both CT and MRI scans. Using the normalized algorithm of the Clinical Toolbox, each individual's cranial scan and lesion locations were transformed to the T1 template, based on older individuals, with a resampled voxel size of 1 × 1 × 1 mm^3^. The precision of spatial normalization was verified by a visual inspection of the standard templates.

Next, we conducted a VLSM analysis with nonparametric mapping^33^ to determine which lesion location(s) were related to sensory phenotypes (as assessed using the bedside-QST) (Fig. 1). To ensure adequate statistical power, voxels with a low probability of damage of 20% or less across all patients were excluded from the statistical analysis, as suggested by past research and guidelines for lesion–symptom mapping.^48,49^ Therefore, the voxels examined in the statistical analysis included regions affected in groups with and without pain. Statistical analysis was performed using the Liebermeister nonparametric tests for examining binomial data (sensory abnormal or normal). This analysis gives a more accurate probability of an event occurring by chance; it can offer better sensitivity, similar to Fisher's exact test.^33^ The permutation-based correction was performed for false positives and negatives to control voxel-level family-wise error that risks false positive voxel (Type 1).^50^ Thus, the family-wise error was adjusted to the significance level via a permutation test (4000 permutations). Three conditions were tested: all patients, CPSP and no-pain, CPSP and non-CPSP. These conditions were assumed because CPSP may strongly lead to sensory abnormality. To visualize lesions, we used external visualization packages such as MRIcroGL (https://www.nitrc.org/projects/mricrogl). Additionally, we used the Automated Anatomical Labelling atlas^51^ and the Johns Hopkins University white-matter tractography atlas^52^ (an anatomical template provided with the MRIcroGL software) as references for anatomical localization.

### Disconnectome analysis

Disconnectome analysis was performed using a Lesion Quantification Toolkit (LQT) to acquire patterns of the white matter disconnection.^53^ The SDC was produced using an LQT after which we conducted an SDC mapping for VDSM after reslicing to the FSL-Montreal Neurological Institute 152 template based on previous research.^53^ VDSM analysis was then performed using the same procedures (see VLSM section).^34^ The detected voxels, which were significantly associated with the abnormal sensation of all patients, CPSP and no-pain, CPSP and non-CPSP, reidentified the disconnected white matter using Human Connectome Project 1065 atlas on MRIcroGL.

### Statistical analysis

Statistical analyses were conducted in R-studio (R V4.2.2). Adequate sample size was determined considering the impact of multiplicity and *post hoc* analyses as confirmed using the G*power tool (Supplementary Fig. 1). To compare each item in the Short Form-McGill Pain Questionnaire-2 between the CPSP and non-CPSP groups, a Mann–Whitney test was used. Variables in the pain questionnaires (NPSI, PDQ, TSK-11 and SF-PCS) and FMA scores were compared between the CPSP, non-CPSP and no-pain groups using a Kruskal–Wallis test with a Bonferroni–Holm *post hoc* correction for multiple comparisons and confirmation of a normal distribution using histograms. The pain-related interview items (joint pain, limited range of motion, mechanical and thermal allodynia, sensory disturbance and subluxation of the shoulder joint), and modified Ashworth scale scores were compared using a χ^2^ test and Fisher's exact test with a Bonferroni–Holm post hoc correction.

For the statistical analysis of bedside-QST, we first translated the variables into binary data using the cut-off values reported in a previous study.^32^ Next, we used the *χ*^2^ test and Fisher's exact test followed by the Bonferroni–Holm correction to compare the variables of hypersensitivity, hypoesthesia and allodynia or hyperalgesia (thermal and mechanical) from the bedside-QST results. Furthermore, multiple logistic regression analysis (Fig. 1B) was used to summarize whether CPSP and non-CPSP were related to these variables based on their statistical significance and the results of previous studies.^12-29,42^ When the logistic regression result was significant, we included up to 7 variables as input, as a previous study indicated that the sample size should be at least 10 times the number of input variables.^54^ Selected variables had values <3 in the variance inflation factor for confirming confounders and multicollinearity. Model fitting was evaluated stepwise using Akaike's information criterion to examine the best relationships with significant variables, and the model was explained using Nagelkerke's R^2^ as the pseudo-coefficient of determination. Odd ratios (ORs), including 95% confidence intervals (CIs) and regression coefficients, were calculated to explain the relationship between the CPSP and non-CPSP groups. Significance was applied at *P* < 0.05.

## Results

In this cross-sectional study, 70 patients were enrolled. Of the 70 patients, 24 had CPSP, 26 had non-CPSP and 20 had no pain. Patient characteristics and demographics are presented in Table 1. The patients in the CPSP group were younger than those in the non-CPSP and no-pain groups, and the poststroke duration also produced similar findings. The pain duration in the CPSP and the non-CPSP groups was not significantly different. While the side of haemiplegia in each group was comparable between the CPSP and the non-CPSP groups, the non-CPSP groups had significantly more left haemiplegia than the no-pain groups. Supplementary Table 1 presents the summary information of lesion and clinical findings. In addition, the differences in ischaemic or haemorrhagic stroke are presented in Supplementary Table 2.

### Bedside-QST in post-stroke pain

**Figure 2:**
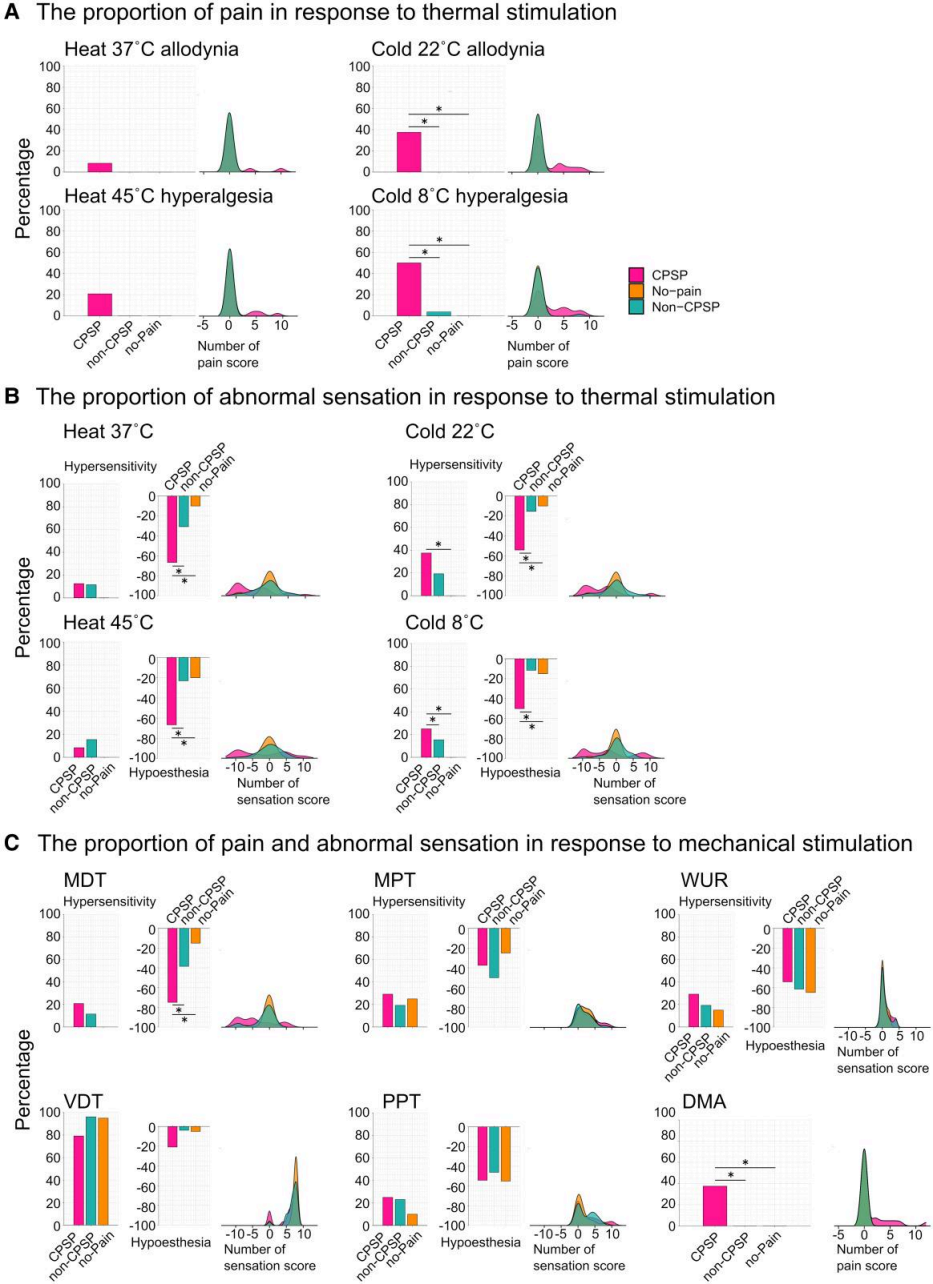
**Comparison of the sensory phenotype in the three subgroups.** (**A**) Comparisons of scores between the subgroups in the bedside-QST, which contain thermal pain parameters. (**B**) Comparisons of scores between the subgroups in the bedside-QST, which contain thermal sensation parameters. (**C**) Comparisons of mechanical sensation parameters. The bar graph indicates the proportions of hypersensitivity and hypoesthesia beyond the cut-off value in the bedside-QST. *P < 0.05, χ^2^ and Fisher's exact tests with a Bonferroni–Holm *post hoc* correction. Deep pink: CPSP; light sea-green: non-CPSP; orange: no-pain. Cold 22°C, innocuous cold stimulus at 22°C; cold 8°C, noxious cold stimulus at 8°C; heat 37°C, innocuous heat stimulus at 37°C; heat 45°C, noxious heat stimulus at 45°C. DMA, dynamic mechanical allodynia; MDT, mechanical detection threshold (sensitivity); MPT, mechanical pain threshold (sensitivity); PPT, pressure pain threshold (sensitivity); QST, quantitative sensory testing; VDT, vibration detection threshold (sensitivity); WUR, wind-up ratio.

Figure 2A shows the proportion of patients who experienced thermal allodynia and hyperalgesia (cold 8C/22°C and heat 37°C/45°C) in the bedside-QST. A greater proportion of CPSP patients compared with the non-CPSP and no-pain groups experienced pain intensity in response to a cold stimulus at 22°C (38%, 0% and 0%, respectively; *P* < 0.05) and 8°C (50%, 4% and 0%, respectively; *P* < 0.05). However, pain intensity in response to a heat stimulus at 37°C/45°C did not significantly differ between the three groups. Figure 2B demonstrates the proportion of patients with hypersensitivity and hypoesthesia in response to heat and cold stimuli. A greater proportion of CPSP patients had cold hypersensitivity in response to the cold stimulus at 22°C compared with the no-pain group (37.5% versus 0%, *P* < 0.05). In addition, a greater proportion of CPSP patients compared with patients in the non-CPSP and no-pain groups had cold hypoesthesia in response to the cold stimulus at 22°C (54.2%, 15.4% and 10%, respectively; *P* < 0.05) and 8°C (50%, 11.5% and 15%, respectively; *P* < 0.05) whereas the proportion of patients with hypersensitivity in response to a cold stimulus at 8°C did not significantly differ between the three groups. Furthermore, a greater proportion of CPSP patients compared with the non-CPSP and no-pain groups also had heat hypoesthesia in response to the heat stimulus at 37°C (66.6%, 30.7% and 10%, respectively; *P* < 0.05) and 45°C (67%, 23% and 20%, respectively; *P* < 0.05).

Figure 2C shows abnormal sensations in response to mechanical stimuli. In response to mechanical stimuli, a greater proportion of CPSP patients compared with the non-CPSP and no-pain groups had touch hypoesthesia (75%, 38.5% and 15%, respectively; *P* < 0.05) and dynamic mechanical allodynia (37.5%, 0% and 0%, respectively; *P* < 0.05). Other parameters were not significantly different between the groups.

### Clinical features of CPSP and non-CPSP

CPSP patients had higher pain quality of Hot-burning, Tender, Cold-freezing, and Numbness in Short Form-McGill Pain Questionnaires-2 (Supplementary Fig. 2).

**Figure 3:**
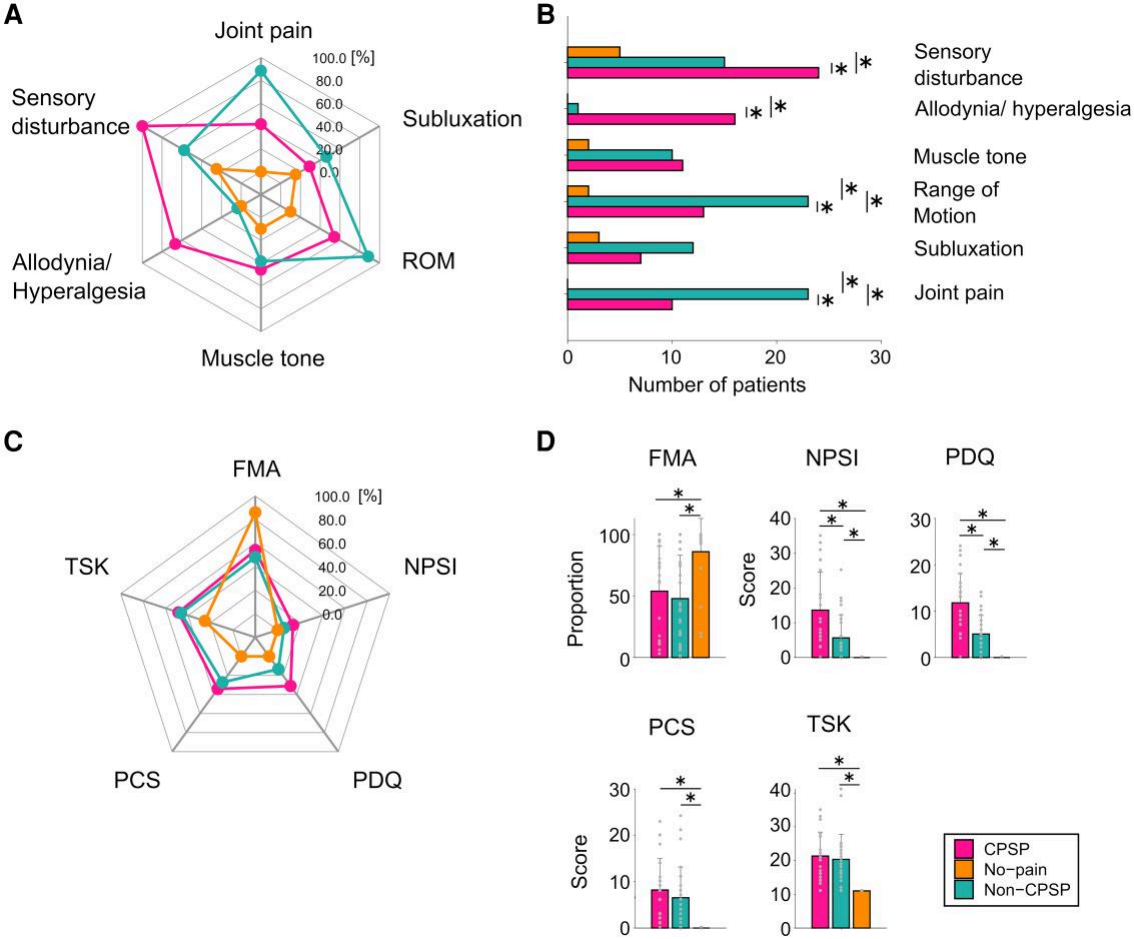
**The difference in the clinical features in the CPSP, non-CPSP and no-pain groups**. (**A and C**) A radar chart showing the difference between the subgroups of each pain-related factor containing neuropathic and nociceptive elements (indicating the proportion of the total score). (**B**) The bar graph shows the comparisons between the subgroups of each pain-related element (*P < 0.05, χ^2^ and Fisher's exact tests with a Bonferroni–Holm *post hoc* correction). (**D**) The bar graph shows the comparisons between the subgroups of each pain-related assessment (the Kruskal–Wallis test with a Bonferroni–Holm *post hoc* correction). The graphs show the means ± SDs. FMA, Fugl–Meyer assessment; NPSI, Neuropathic Pain Symptom Inventory; PCS, Short-Form Pain Catastrophizing Scale; PDQ, Pain Detection Questionnaire; ROM, range of motion; TSK-11, Tampa Scale for Kinesiophobia-11.

Figure 3A and C show the visualization of differences in clinical features between the CPSP, non-CPSP and no-pain groups. As shown in Fig. 3B, a greater proportion of CPSP patients compared with the non-CPSP and no-pain groups had somatosensory disturbance (100%, 57.7% and 25%, respectively; *P* < 0.05) and allodynia (thermal and mechanical; 66.7%, 3.8% and 0%, respectively*; P* < 0.05). By contrast, a greater proportion of non-CPSP patients had range of motion limitations (non-CPSP 88.4%, CPSP 54.1%, no-pain 10%; *P* < 0.05) and joint pain triggered by movement (non-CPSP 88.4%, CPSP 41.6%, no-pain 0%; *P* < 0.05) compared with the other two groups. The proportions of patients with abnormal muscle tone and subluxation did not differ significantly between the three groups. Figure 3D shows the NPSI, PDQ, SF-PCS, TSK-11 and FMA results. The NPSI scores of CPSP patients were higher than those of non-CPSP and no-pain patients (13.6% ± 10.6%, 5.6% ± 6.5% and 0, respectively; *P* < 0.05). In addition, the NPSI scores of non-CPSP patients were higher than those of no-pain patients. The PDQ scores of CPSP patients were higher than those of non-CPSP and no-pain patients (11.8 ± 6.2, 5.1 ± 3.9 and 0, respectively; *P* < 0.05). Similarly, the PDQ scores of non-CPSP patients were higher than those of no-pain patients (*P* < 0.05). The TSK-11 scores of CPSP and non-CPSP patients were higher than those of no-pain patients (21.3 ± 6.9, 20.3 ± 7.2 and 11 ± 0, respectively; *P* < 0.05). The SF-PCS scores of CPSP and non-CPSP patients were higher than those of no-pain patients (8.2 ± 6.6, 6.5 ± 6.4 and 0, respectively; *P* < 0.05). Moreover, the FMA scores of no-pain patients were higher than those of CPSP and non-CPSP patients (86.1% ± 26.3%, 53.9% ± 35.5% and 47.9% ± 34.5%, respectively; *P* < 0.05).

### Multiple logistic regression analysis

**Figure 4:**
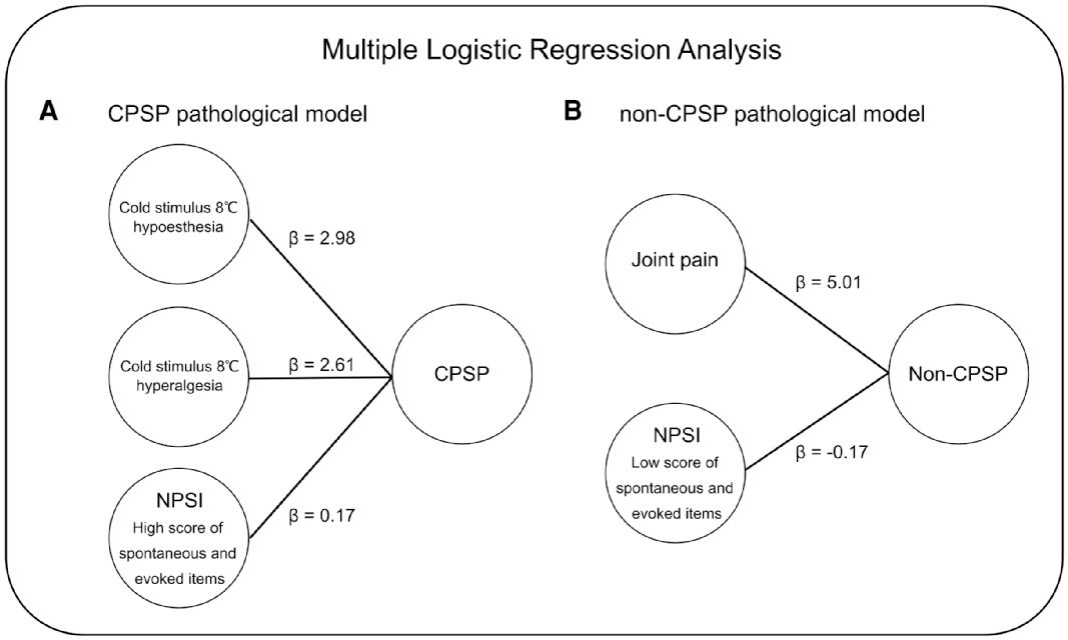
**Relationships between elements or clinical features and CPSP or non-CPSP**. (**A**) The graph indicates the relationships between each element related to neuropathic pain and the presence of CPSP. (**B**) The graph indicates the relationships between each element related to nociceptive pain and the presence of non-CPSP. The relationship between CPSP or non-CPSP and each element represented by the black line indicating β in the multiple logistic regression. CPSP, central post-stroke pain; NPSI, Neuropathic Pain Symptom Inventory.

The variance inflation factors in the CPSP model were 1.08 for both cold hypoesthesia at 8˚C and the NPSI. The variance inflation factors in the non-CPSP model were 1.0 for each of joint pain and spontaneous and evoked pain in the NPSI (Table 2). The model fitting was explained as Akaike's information criterion 44.47 in the CPSP model and Akaike's information criterion 52.53 in the non-CPSP model (Supplementary Tables 3 and 4). As shown in Fig. 4A (left panel), CPSP had a significant relationship with presence of hypoesthesia (β = 2.98, OR = 19.6, 95% CI = 2.7–141.8) and hyperalgesia at cold 8°C (β = 2.61, OR = 13.6, 95% CI = 1.13–163.12) and high the sum score of spontaneous and evoked pain items in the NPSI (β = 0.17, OR = 1.19, 95% CI = 1.07–1.32); the model was explained by 75.6% in the Nagelkerke analysis (Table 2). In contrast, non-CPSP had a significant relationship with presence joint pain (β = 5.01, OR = 149.854, 95% CI = 19.93–1126.52) and low the sum score of spontaneous and evoked pain items in the NPSI (β = −0.17, OR = 0.8, 95% CI = 0.75–0.94; Fig. 4B right panel); the model was explained by 65.5% in the Nagelkerke analysis (Table 2).

**Table 2:** Relationships between each element and CPSP or non-CPSP

	*β*	Std. Error	*P*-value	Odd	95% CI	VIF	AIC	Nagelkerke R2 (%)
CPSP model								
cold22 allodynia	18.94	2813.22	0.99	1.68E + 0		1.00	44.47	75.6
cold8 hyperalgesia	2.61	1.27	0.03	13.6	1.134–163.12	1.02		
cold8 hypoesthesia	2.98	1.01	0.003	19.6	2.7–141.8	1.32		
High score of NPSI (spontaneous and evoked pain)	0.17	0.05	0.0009	1.19	1.07–1.32	1.29		
Non-CPSP model								
Joint pain	5.01	1.03	0.0000011	149.84	19.93–1126.52	1.73	52..53	65.5
Low score of NPSI (spontaneous and evoked pain)	−0.17	0.06	0.003	0.804	0.75–0.94	1.73		

Cells show the parameter estimate, standard error, *P*-value, odds ratio, and confidence interval. The pseudo-R2 value in each model was calculated using Nagelkerke's method.

CI, confidence interval; Odd, odds ratio; std.error, standard error; VIF, variance inflation factor.

### Brain lesion analysis

**Figure 5:**
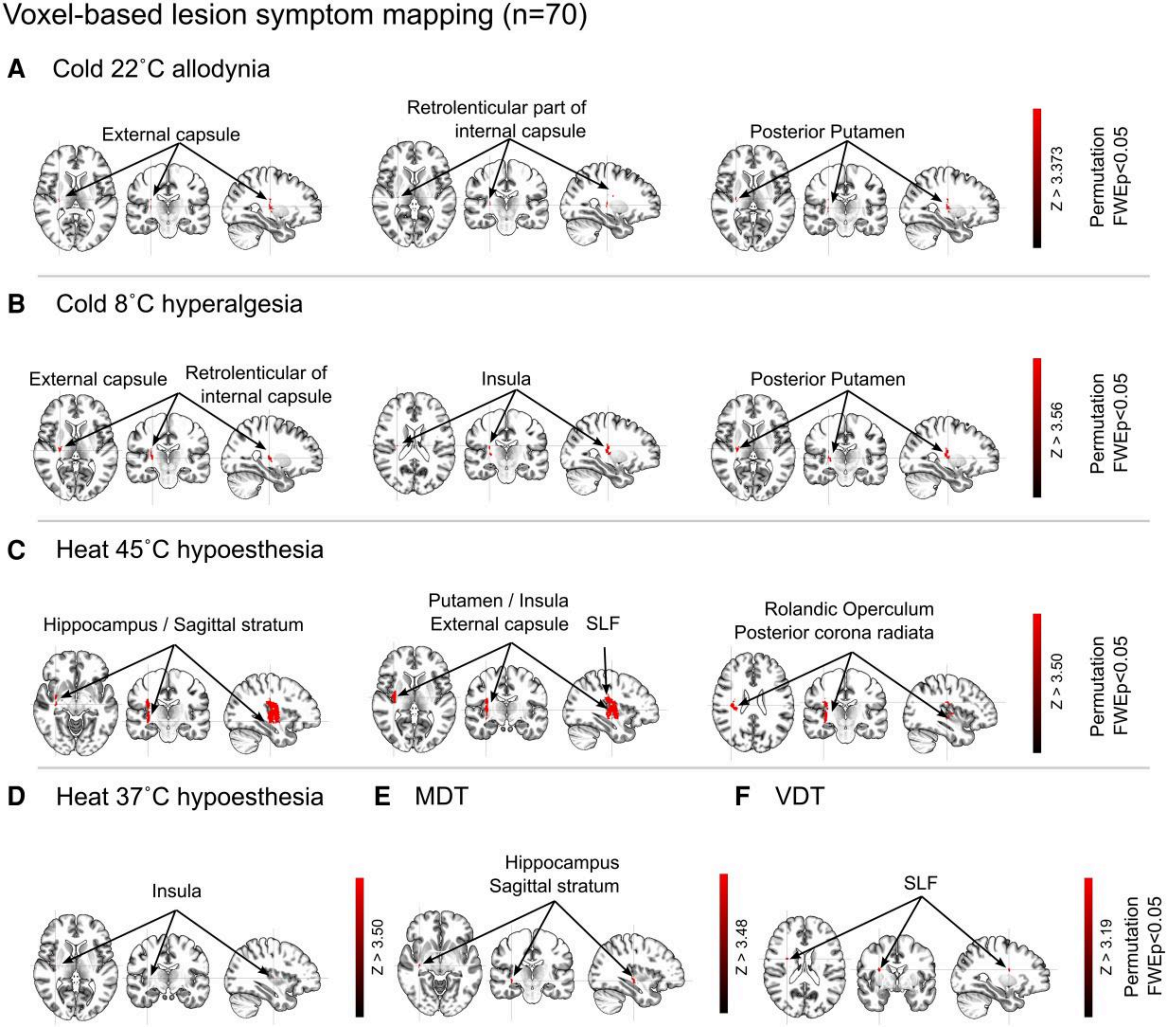
**Anatomical results obtained from the VLSM analyses in all patients (CPSP, non-CPSP and no-pain).** (**A–F**) The VLSM results indicate the areas associated with significant items in the bedside-QST among all patients (only voxels involving at least 14 patients were included in VLSM). The colour bars represent *Z* scores; colour-highlighted voxels are expressed at above-threshold regions (cold allodynia 22°C: *Z* = 3.73; cold hyperalgesia 8°C: *Z* = 3.56; heat hypoesthesia at 45°C: *Z* = 3.5; MDT: *Z* = 3.48; heat hypoesthesia at 37°C: *Z* = 3.5; VDT: *Z* = 3.19). The lesion map in the VLSM presents only the right side (FDR, *P* < 0.05, permutation family-wise error, *P* < 0.05). CPSP, central post-stroke pain; FDR, false discovery rate; FWE, family-wise error; MDT, mechanical detection threshold (sensitivity); QST, quantitative sensory testing; SLF, superior longitudinal fasciculus; VDT, vibration detection threshold (sensitivity); VLSM, voxel-based lesion–symptom mapping.

Figure 5 demonstrates the VLSM results regarding the relationship between bedside-QST scores and areas of brain lesions in all patients. The extracted areas indicated that voxels were significantly associated with cold hyperalgesia and cold allodynia at 8°C (*Z* > 3.56) and 22°C (*Z* > 3.37), heat hypoesthesia at 37°C (*Z* > 3.5) and 45°C (*Z* > 3.5; Fig. 5A–D). In addition, the areas also indicated that voxels were significantly associated with hypoesthesia at touch and vibration detection threshold (VDT; Fig. 5E and F; see Supplementary Table 5). Areas associated with cold allodynia and hyperalgesia were detected in the posterior putamen, insula, external capsule and the retrolenticular part of the internal capsule (x, y and z of voxel volume, see Supplementary Table 5). Areas associated with heat hypoesthesia were detected in the insula, putamen, Rolandic operculum, hippocampus, posterior corona radiata, superior longitudinal fasciculus (SLF), sagittal stratum and external capsule. In addition, the area associated with the other parameters (touch and VDT) was detected in similar areas (Supplementary Table 5).

Supplementary Fig. 3A–F demonstrates the areas that correlated with bedside-QST data in brain images of the CPSP and no-pain groups (*n* = 44). The extracted areas indicated that voxels were significantly associated with cold hyperalgesia and cold allodynia at 8°C (*Z* > 3.57) and 22°C (*Z* > 3.51), and heat hypoesthesia at 45°C (*Z* > 3.59) and 37°C (*Z* > 3.54) (Supplementary Table 6). Areas associated with cold allodynia and hyperalgesia were detected in the posterior putamen, insula, external capsule and superior corona radiata. Areas associated with heat hypoesthesia at 45°C were the putamen, insula, Rolandic operculum, hippocampus, external capsule, SLF and sagittal stratum. Similarly, areas associated with heat hypoesthesia at 37°C were only the superior corona radiata. The touch and VDT were also detected in similar areas (Supplementary Table 6).

Supplementary Fig. 4A–D demonstrates the areas that correlated with bedside-QST data in brain images of the CPSP and non-CPSP groups (*n* = 50). The extracted areas indicated that voxels were significantly associated with cold hyperalgesia and cold allodynia at 8°C (*Z* > 3.58) and 22°C (*Z* > 3.5), heat hypoesthesia at 45°C (*Z* > 3.59) and the wind-up ratio with pinprick stimulus (*Z* > 3.53; Supplementary Table 7). Areas associated with cold allodynia and hyperalgesia at 8°C and 22°C were the posterior putamen, external capsule and retrolenticular part of the internal capsule. Areas associated with heat hypoesthesia at 45°C were the insula, putamen, Rolandic operculum, external capsule and SLF. Areas associated with the wind-up ratio were the anterior insula and the SLF, whereas the areas that correlated with bedside-QST data in brain images of patient participants in the no-pain and non-CPSP groups (*n* = 46) were only significantly associated with heat hypoesthesia at 45°C (see Supplementary Table 8 and Supplementary Fig. 5).

### Disconnectome analysis

**Figure 6:**
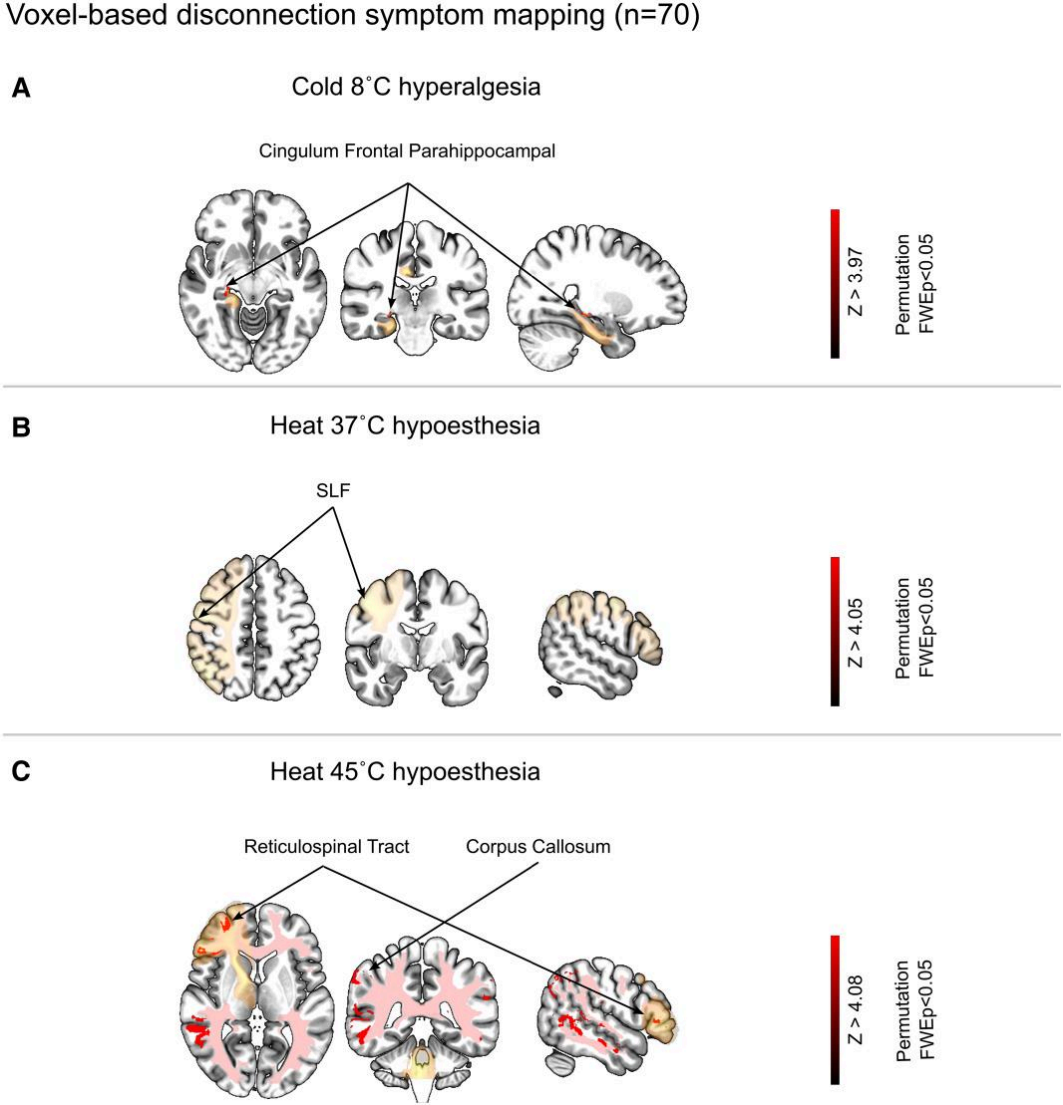
**The results of VDSM in all patients (CPSP, non-CPSP and no-pain).** (**A–C**) The VDSM results indicate disconnection of white matter associated with significant items in the bedside-QST among all patients (20% of the sample number). The colour bars represent *Z* scores; colour-highlighted disconnection is shown at above-threshold regions (cold hyperalgesia at 8°C: *Z* = 3.97; heat hypoesthesia at 37°C: *Z* = 4.05; heat hypoesthesia at 45°C: *Z* = 4.08). The disconnection map in the VDSM presents intrahemispheric and interhemispheric disconnections (FDR, *P* < 0.05, permutation family-wise error, *P* < 0.05). CPSP, central post-stroke pain; FDR, false discovery rate; FWE, family-wise error; SLF, superior longitudinal fasciculus; VDSM, voxel-based disconnection–symptom mapping.

Figure 6 demonstrates the VDSM results regarding the relationship between bedside-QST scores and patterns of white matter disconnection in all patients. The extracted disconnection maps were significantly associated with cold hyperalgesia at 8°C (*Z* > 3.97) and heat hypoesthesia at 37°C (*Z* > 4.05) and 45°C (*Z* > 4.08; Fig. 6A–C; Supplementary Table 5). The peak *Z* scores in the family-wise error (FWE)-corrected VDSM map associated with cold allodynia indicated involvement of the cingulum frontal parahippocampal region (Fig. 6A). For heat hypoesthesia at 37°C, the peak *Z* scores were associated with the SLF (Fig. 6B). Additionally, the peak Z scores for heat hypoesthesia at 45°C involved the reticulospinal tract and a widespread interhemispheric disconnection of the corpus callosum (Fig. 6C). The other parameters detected with VLSM were not significantly disconnected white matter (Supplementary Table 5).

The disconnection maps implicated in the CPSP and no-pain groups were significantly associated with heat hypoesthesia at 37°C (*Z* > 4.07) and 45°C (*Z* > 4.00), hypoesthesia at mechanical detection threshold (MDT; *Z* > 4.00) and VDT (*Z* > 3.62; Supplementary Figs 6A–D; Supplementary Table 6). The peak *Z* scores in the family-wise error (FWE)-corrected VDSM map associated with heat hypoesthesia at both 37°C and 45°C involved the corpus callosum and the thalamic radiata superior across the thalamus to frontal areas (Supplementary Fig. 6A and B). In addition, the disconnection of white matter associated with heat hypoesthesia at 45°C only involved the corticothalamic pathway. The peak *Z* scores for hypoesthesia at MDT involved inferior longitudinal fasciculus, while the peak *Z* score for hypoesthesia at VDT involved in the reticulospinal tract and cingulum frontal–parietal bundle.

The disconnection maps implicated a significant association with heat hypoesthesia at 45°C in two conditions: the CPSP and non-CPSP (*Z* > 4.03) and the no-pain and the non-CPSP (*Z* > 3.88; Supplementary Tables 7 and 8). The peak *Z* scores of VDSM maps in the CPSP and non-CPSP groups involved the arcuate fasciculus, while the peak *Z* scores of VDSM maps in the no-pain and non-CPSP groups indicated the anterior thalamic radiata (Supplementary Figs 7 and 8; Supplementary Tables 7 and 8).

## Discussion

In the present study, we aimed to identify the clinical features and pathologies of PSP using comprehensive assessments. Here, we will discuss these features and pathologies based on the results of our clinical assessments and brain lesion analysis.

### Clinical features of CPSP and Non-CPSP patients

CPSP patients in the present study had higher pain intensity in Hot-burning, Tender, Cold-freezing and Numbness (Supplementary Fig. 2). These descriptions were similar to those from previous studies of CPSP patients.^5,7,8,13^ Our data also indicated that abnormal heat/cold pain, mechanical allodynia and numbness were CPSP-specific pain qualities. Furthermore, in the present study, CPSP patients had somatosensory disturbances and symptoms of neuropathic pain (i.e. higher NPSI and PDQ scores) in pain questionnaires and basic clinical assessments. In contrast, non-CPSP patients had the lowest motor function, a limited range of motion and joint pain triggered by movement. These clinical features of CPSP and non-CPSP patients are comparable with those reported in previous studies.^12-16,19-21,26-29,42^ In the bedside-QST, CPSP patients had abnormal responses to not only thermal stimulation but also tactile stimulation; that is, thermal and mechanical hypoesthesia, cold allodynia, cold hyperalgesia and dynamic mechanical allodynia were observed in CPSP patients. In particular, dynamic mechanical allodynia and abnormal responses to an innocuous stimulus at 22°C and a noxious stimulus at 8°C were specific to CPSP. Studies have reported that abnormal temperature sensation and mechanical allodynia can be caused by injury to part of the spinothalamic pathway.^14,55,56^ Meanwhile, the non-CPSP and no-pain did not show completely abnormal sensations in the thermal and mechanical tests (Fig. 2). These observations indicate that these groups did not have abnormal sensations consistent with neuropathic pain. Notably, the no-pain groups had brain injuries, yet we considered that the QST demonstrated typical values, suggesting no abnormalities in the somatosensory system. We demonstrated that the primary difference in abnormal sensation between CPSP and non-CPSP patients was hypoesthesia in response to both innocuous stimuli (cold at 22°C, heat at 37°C and MDT) and noxious stimuli (cold at 8°C and heat at 45°C). Multiple logistic regression analysis revealed that CPSP was characterized by cold hyperalgesia and hypoesthesia at 8°C, as well as high NPSI scores. This result suggests that in addition to noxious cold hyperalgesia and hypoesthesia, tending high spontaneous and evoked pain are characteristic features of CPSP. Although non-CPSP patients had no specifically abnormal sensations, the existence of musculoskeletal pain factors was characteristic of non-CPSP. Particularly, non-CPSP was characterized by the existence of joint pain and low NPSI scores. These findings contrast with those of CPSP; non-CPSP is characterized mainly by musculoskeletal factors without neuropathic pain symptoms. Considering that cold hypoesthesia and hyperalgesia are reported as typical symptoms in neuropathic pain, whereas movement-triggered joint pain reportedly comes from musculoskeletal problems such as tissue injury and shortening muscle arises from immobility or increasing muscle tone with spasticity, these results are convincing. Furthermore, although it is difficult to draw definite conclusions, the higher prevalence of left haemiplegia in the non-CPSP groups may be linked to tissue injury caused by limb positioning due to sensory deficits or agnosia.^26,57^ Together, our findings confirm and indicate that these clinical features are key for distinguishing CPSP from non-CPSP. Although these findings are not novel, they support the findings of previous studies, and are important indicators to facilitate decision-making or optimize the precision treatments by data dimensionality reduction when making a diagnosis using clinical assessments, such as the bedside-QST, pain-related factor and pain questionnaires.

### Neural correlations with sensory phenotypes in CPSP

We analysed the relationships between somatosensory features and brain lesion areas using VLSM analysis. In addition, we also compared somatosensory features and the lesion-induced SDC using VDSM analysis. An analysis of all patients (Fig. 5) demonstrated that hyperalgesia and allodynia in response to both noxious and innocuous cold stimuli at 8°C and 22°C correlated with lesions of the posterior putamen, insula, external capsule and the retrolenticular part of the internal capsule. In addition, the VDSM analysis of all patients (Fig. 6) demonstrated that cold hyperalgesia in response to noxious cold stimuli at 8°C was corrected with the cingulum frontal parahippocampal tract. When we analysed VLSM with imaging data from the CPSP and no-pain groups alone (Supplementary Fig. 3), the posterior putamen, insula, external capsule and superior corona radiata were also correlated with cold hyperalgesia and allodynia. Similarly, when we analysed imaging data from the CPSP and non-CPSP groups alone (Supplementary Fig. 4), similar results were obtained (in addition to the aforementioned lesion areas except insula and superior corona radiata, the retrolenticular part of internal capsule correlated with cold allodynia and hyperalgesia). Although these lesion areas have been reported to cause CPSP,^17,18,20,56-58^ previous studies have reported the frequency of lesions diagnosed from brain imaging findings but have not included objective assessments. Therefore, it was unclear whether the findings were necessarily related to behavioural data (e.g. even with the same thalamic lesion, some patients may present with abnormal sensations or pain, while others do not). In addition, previous studies have barely reported structural disconnection patterns regarding the abnormal sensation caused by CPSP. Considering this background, the objective extraction of the relationship between brain lesion areas and clinical findings in the present study is novel and clinically important. Our study revealed that lesions of the posterior putamen and insula may be associated with cold allodynia and hyperalgesia after stroke. Furthermore, results from those with lesions of white matter fibres also demonstrated that the external capsule and retrolenticular part of the internal capsule correlated with cold allodynia and hyperalgesia. The lateral spinothalamic tract passes through these lesion areas,^17,18,58^ indicating that lesions of the lateral spinothalamic tract led to cold allodynia and hyperalgesia. Particularly, cold hyperalgesia may be associated with these areas because cold at 8°C is processed via the lateral spinothalamic tract. In addition, the retrolenticular part of the internal capsule involves the posterior portion of the putamen and the dorsal part of the posterior limb of the internal capsule, just adjacent to the thalamus, where the ascending sensory pathway occurs.^56^ Considering that the thalamus, insula, putamen and retrolenticular and posterior parts of the internal capsule are important areas for the pain matrix and thermal perception,^3,14,17,18,56-58^ the relationship between cold allodynia or hyperalgesia and lesions in these areas is not surprising. By contrast, the result of VDSM analysis implicated the cingulum frontal parahippocampal tract, which connects the insula, the frontal areas to the orbital frontal cortex, the anterior cingulate cortex and the hippocampus via the cingulum bundle. These areas have been found to involve the reward or emotion in the pain processes,^59,60^ namely, reflected in the medial pain pathway composed of the spinoreticular and the spinotectal tracts.^55^ Although the result of VDSM and VLSM analysis is inconsistent with these pain processing systems, considering the disconnection induced from the lesion, the lesion may cause the lateral spinothalamic pathway with the constructive issue and disturb the medial spinothalamic pathways. By contrast, in non-CPSP patients, no clear correlation was found between the lesion and the disconnection with abnormal thermal perception. Moreover, a detailed analysis of sensory function (both gain and loss) and lesion mapping in individual patients revealed that, despite similar lesions, only CPSP patients developed pain, whereas non-CPSP and no-pain patients did not (Supplementary Tables 1 and 2). Differences might result from white matter affected by a broadening lesion (particularly those affected by haemorrhage stroke) in the structure with the lesion. We consider that the non-CPSP might not be related to the brain lesion because patients with non-CPSP often have damage in the internal capsule, temporal and parietal areas, which might not correlate with their clinical characteristics.

Hypoesthesia with a noxious heat stimulus at 45°C correlated with lesions of the insula, putamen, Rolandic operculum, hippocampus, posterior coronal radiata, SLF, sagittal stratum (SS) and external capsule in the analysis of all patients (Fig. 5). When we also analysed VLSM with imaging data from the patients in the CPSP and no-pain groups, or the CPSP and non-CPSP groups (Supplementary Figs 3C and 4D), similar lesions were correlated with hypoesthesia with a noxious heat stimulus at 45°C. We specifically found that the putamen, external capsule and insula are common areas across the three comparison conditions. Furthermore, the non-CPSP and no-pain also were extracted in the same areas (Supplementary Fig. 5). These common areas are known as the salience networks (SN) involving with pain processing (multiple sensory interaction) in a previous study.^61^ In other words, the SN may be reduced when there is a loss of function from heat stimulation at 45°C. However, this phenomenon may be commonly associated with the loss of thermal perception after stroke because the non-CPSP and no-pain were also correlated with similar stimuli.^62,63^ Meanwhile, the Rolandic operculum, hippocampus, SLF, posterior corona radiata and SS were specifically associated with abnormal sensation in response to heat stimuli in patients in the CPSP group but not those in the non-CPSP and pain groups. Previous studies have reported that lesions between the posterior insula and operculum cause pain and abnormal perception.^16,25^ Moreover, an important finding of the present study was that cold allodynia in response to a cold stimulus when there were lesions of the retrolenticular portion of the posterior limb of the internal capsule, whereas heat hypoesthesia in response to a noxious heat stimulus at 45°C correlated with lesions of the posterior insula and Rolandic operculum. These areas are related to the thalamus–insula–operculum projection, which is particularly connected with the posterior part of the ventral medial nucleus and the insula. These lesions are considered affected by thalamocortical interruptions that develop CPSP.^55,58^ The SLF and posterior corona radiata are involved in sensory integration and pain perception.^64^ In particular, the SLF generally links the frontal, parietal and temporal lobes involving pain, emotion and sensory processes. Pain and abnormal thermal perception in CPSP may therefore be associated with lesions in projections between the subcortical and secondary somatosensory cortex.^16,25,58^ In addition to these areas, the hippocampus confirmed with the specific area by a noxious heat stimulus at 45°C and the insula confirmed with the common area by a noxious heat at 45°C and cold at 8°C, which are related to the pain matrix and the descending pain modulation system. When lesions in these areas are considered, the patients in the CPSP group may not only have dysfunction in the ascending pain pathways but also impairments in descending pain modulation. This idea can be confirmed with the reticulospinal tract associated with the heat stimulus at 45°C of the VDSM result (Fig. 6), and it involves pain inhibition because this tract ultimately reaches the periaqueductal gray matter. Therefore, lesions and connectome in these areas might lead to an imbalance between cold and heat input, or between the ascending and descending pathways, thus causing CPSP.^14,55,65^ In contrast with the results of the VLSM analysis, hypoesthesia with a noxious heat stimulus at 45°C and an innoxious heat stimulus at 37°C correlated with the corpus callosum in the VDSM analysis (Fig. 6 and Supplementary Fig. 6). Although the involvement of this fibre is consistent with the VLSM results for pain processing, it also plays a role in interhemispheric connectivity. Additionally, it is associated with nociceptive thresholds and sensory processing.^66^ Furthermore, a previous study has identified this fibre as important for neuropathic pain processing.^64^ The corpus callosum contributes to sensory integration within default mode networks (DMN),^67^ and heat hypoesthesia at 45°C and 37°C, which may indicate reduced sensory integration and a lowered pain threshold in the DMN, potentially enhancing or suppressing interhemispheric connectivity. These findings must precisely support indicating more relevance between CPSP and abnormal somatosensory (thermal) sensation.

The mechanical stimuli (MDT and VDT) correlated with lesions in the sagittal stratum, SLF and hippocampus in the VLSM analysis of all patients (Fig. 5E and F). When we analysed the CPSP and no-pain groups, we found similar areas (Supplementary Fig. 3). Although CPSP was not typically associated with the somatic sensory system, the lesions identified in this study differed from those usually associated with pure sensory abnormalities. The hippocampus also plays a role in affective pain processing. Moreover, the SS provides connectivity between multiple lobes, including the parietal and temporal lobes. These systems compose the SN^61^ and may be associated with disturbed pain cognitive processing in the hypoesthesia at MDT and VDT, which appears as CPSP. The lesion in the hippocampus and sagittal stratum associated with abnormal sensation in response to MDT might specifically demonstrate noxiousness. Similarly, VDSM analysis also highlighted the SS, including the inferior longitudinal fasciculus. Additionally, when we analysed imaging data from the CPSP and non-CPSP groups alone, lesions in the insula cortex and the SLF were correlated with wind-up in the VLSM analysis. However, the VDSM was not extracted statistically. These findings indicate that the lesion areas correlated with the wind-up were extracted among CPSP and non-CPSP. Considering that the wind-up ratio demonstrates symptoms of central sensitization, the aforementioned lesion areas may likely be more sensitive responses to pain.^68^ Similarly, the results of a previous study suggest that the insula and putamen are key areas for central sensitization.^69^ Furthermore, these areas are important both in the pain matrix and for emotional functions; previous studies have reported that central sensitization is associated with emotional aspects.^55,70^ The German Research Network on Neuropathic Pain reported that wind-up is a surrogate marker of central sensitization.^31^ In the present study, wind-up was associated with lesions in the anterior insula. Considering that the insula is a key structure for the pain matrix, central sensitization associated with insula lesions would be associated with sensory-processing problems to pain.^68,70^

### Conclusions and limitations

The present study aimed to capture the varied pathological features of PSP. Our findings indicate that CPSP patients have pain and abnormal sensations caused by cortical and subcortical lesions, whereas non-CPSP patients have peripheral tissue problems. The present study is limited because some findings lack novelty and do not represent a significant developmental advance over previous studies. Nevertheless, optimizing the multidimensional phenotype can thus lead to efficient or more precise treatments. In addition, several patients had mixed pain, which was suspected to be neuropathic and nociceptive. This limitation was difficult to unravel in the present study and requires more detail to dissect each pathology. Moreover, the patient sample in this study cannot be said to have no sensory symptoms at all, as it is possible they had not noticed small abnormal sensations from a previous stroke. We cannot determine the mechanism for each pathology because this study was cross-sectional and not longitudinal, so the study results should be interpreted with caution. A longitudinal study is required to interpret the mechanism process translating the chronic phase and each pathology. In addition, the results of the present study are limited by the relatively small sample size. Hence, future studies with larger sample sizes are required to confirm the findings of sensory features and the brain lesions related to abnormal sensation while following the symptoms in each pathology. In imaging analysis, we considered the relationship between the brain image and the NPSI or the SF-MPQ but did not find pain-related brain lesions or disconnection. However, pain pathologies are associated with several factors; thus, these factors also warrant investigation in detail, including the emotional factor (e.g. the SF-MPQ affective dimension) and its association with pain-related lesions. Future studies are necessary to further subdivide the pathology of PSP and elucidate the brain network functions associated with CPSP.

## Supplementary Material

fcaf128_Supplementary_Data

## Data Availability

The data that support the findings of this study are available from the corresponding author, upon reasonable request.
