# Supplementary material for: Pathological features of post-stroke pain: a comprehensive analysis for subtypes
Source: Brain Commun. 2025 Apr 30;7(3):fcaf128. doi: 10.1093/braincomms/fcaf128 (PMC12042915; doi:10.1093/braincomms/fcaf128)
Supplement: fcaf128_Supplementary_Data [file fcaf128_supplementary_data.pdf]

Supplemental Material

Supplementary Questionnaire: Interview sheet for pain-related factor assessment 2

Supplementary Table 1: Summarized data table for individual patients 3-15

Supplementary Fig 1: Sample Size (used G*power) 15

Supplementary Table 2: Comparison of outcomes in CH and CI 16-17

Supplementary Fig 2: Characteristics of pain and pain quality in CPSP and non-CPSP patients 18

Supplementary Table 3: CPSP model variable selected for Akaike's information criteria (AIC)........... 19-22

Supplementary Table 4: Non-CPSP model variable selected for Akaike's information criteria (AIC) 23

Supplementary Table 5: Statistical ratio and brain areas in the CPSP, non-CPSP, and no-pain groups. 24-26

Supplementary Fig 3 and Fig 6: The results of VLSM and VDSM in CPSP and no-pain groups.......... 27-30

Supplementary Table 6: Statistical ratio and brain areas in the CPSP and no-pain groups 31-33

Supplementary Fig 4 and Fig.7: The results of VLSM and VDSM in CPSP and non-CPSP groups...... 34-36

Supplementary Table 7: Statistical ratio and brain areas in the CPSP and non-CPSP groups................ 37-38

Supplementary Fig 5 and Fig.8: The results of VLSM and VDSM in no-pain and non-CPSP groups... 39-40

Supplementary Table 8: Statistical ratio and brain areas in the no-pain and non-CPSP groups...................41

Supplementary Questionnaire

Interview sheet for pain-related factor assessment

1. Whether the pain is triggered by joint movement Yes/No
2. Does the patient have a subluxation on the hemiplegic shoulder? Yes/No
3. Does the patient have a restricted range of motion? Yes/No
4. Does the patient have allodynia or hyperalgesia? Yes/No
5. Does the patient have a sensory disturbance? (using monofilaments) Yes/No

Supplementary Table 1

Summarized data table for individual patients

Patients ID	Groups	Stroke Type	Main Injury Areas	Durations until image measurement day from onset day
1	CPSP	CH	Putamen	25
2	CPSP	CH	Putamen	240
3	CPSP	CH	Putamen	7
4	CPSP	CH	Thalamus	1
5	CPSP	CH	Thalamus	2594
6	CPSP	CI	Inner medulla oblongata	1
7	CPSP	CI	Lateral Thalamus	215
8	CPSP	CH	Thalamus	0
9	CPSP	CI	Brainstem	5
10	CPSP	CI	Frontal, parietal, and temporal areas	5
11	CPSP	CH	Frontal areas	0
12	CPSP	CI	Insula, Operculum, Putamen, Heschl's gyrus	169
13	CPSP	CI	Parietal areas	80
14	CPSP	CH	Parietal areas	31
15	CPSP	CH	Putamen	75
16	CPSP	CH	Thalamus	9
17	CPSP	CI	Pons	23
18	CPSP	CI	Thalamus	1
19	CPSP	CI	Putamen	5
20	CPSP	CH	Putamen	8
21	CPSP	CI	Lateral medulla oblongata	27
22	CPSP	CH	Thalamus	28
23	CPSP	CH	Thalamus	15
24	CPSP	CH	Thalamus	28
25	Non-CPSP	CI	Inner medulla oblongata	2
26	Non-CPSP	CH	Putamen	63
27	Non-CPSP	CH	Thalamus	11
28	Non-CPSP	CI	Posterior internal capsule-corona radiata	20
29	Non-CPSP	CH	Putamen	2
30	Non-CPSP	CI	Corona radiata	6
31	Non-CPSP	CI	Frontal areas	1
32	Non-CPSP	CI	Corona radiata	4

33	Non-CPSP	CI	Corona radiata-Parietal Areas	0
34	Non-CPSP	CI	Putamen-Corona radiata	0
35	Non-CPSP	CH	Thalamus	0
36	Non-CPSP	CH	Thalamus	224
37	Non-CPSP	CI	Corona radiata	10
38	Non-CPSP	CI	Temporal areas	7
39	Non-CPSP	CI	Temporal and parietal areas, Corona Radiata	278
40	Non-CPSP	CI	Basal ganglia-Corona Radiata	0
41	Non-CPSP	CI	Ventral medulla oblongata	0
42	Non-CPSP	CH	Putamen	28
43	Non-CPSP	CI	Medulla oblongata	1
44	Non-CPSP	CH	Pons and Midbrain	1
45	Non-CPSP	CI	Putamen, Corona radiata, Internal capsule	8
46	Non-CPSP	CH	Thalamus	0
47	Non-CPSP	CI	Internal capsule-Corona radiata	7
48	Non-CPSP	CI	Basal ganglia-Corona Radiata	0
49	Non-CPSP	CH	Thalamus	20
50	Non-CPSP	CI	Temporal-Parietal areas	3
51	No-pain	CH	Thalamus	15
52	No-pain	CI	Cellebellum, Occipital area	0
53	No-pain	CI	Putamen	19
54	No-pain	CI	Corona Radiata	1
55	No-pain	CI	Corona Radiata	2
56	No-pain	CI	Temporal areas	0
57	No-pain	CH	Medial Thalamus	0
58	No-pain	CI	Corona radiata	0
59	No-pain	CH	Parietal areas	1
60	No-pain	CI	Corona radiata	0
61	No-pain	CH	Occipital areas	0
62	No-pain	CH	Internal capsule, Retrolenticular part of internal capsule	4
63	No-pain	CH	Putamen	13
64	No-pain	CI	Medulla oblongata	0
65	No-pain	CI	Putamen	36
66	No-pain	CH	Thalamus	7
67	No-pain	CH	Putamen	30
68	No-pain	CI	Prefrontal areas	6
69	No-pain	CI	Thalamus	0
70	No-pain	CI	Insula, Putamen, Corona radiata	36

			Bedside-QST Thermal pain and sensation			
Patients_ID	Groups	Stroke Type	Heat 37˚C Pain	Heat 45˚C Pain	Cold 22˚C Pain	Cold 8˚C Pain
1	CPSP	CH	0	2	0	6
2	CPSP	CH	4	4	4	5
3	CPSP	CH	0	9	0	2
4	CPSP	CH	0	0	0	0
5	CPSP	CH	0	7	0	4
6	CPSP	CI	0	0	0	9
7	CPSP	CI	0	0	0	0
8	CPSP	CH	10	0	9	0
9	CPSP	CI	0	0	0	0
10	CPSP	CI	0	6	0	0
11	CPSP	CH	0	0	0	0
12	CPSP	CI	0	0	1	0
13	CPSP	CI	0	0	0	0
14	CPSP	CH	0	0	0	0
15	CPSP	CH	0	8	0	8
16	CPSP	CH	0	0	0	2
17	CPSP	CI	0	0	0	0
18	CPSP	CI	0	4	6	5
19	CPSP	CI	0	5	5	8
20	CPSP	CH	0	0	0	0
21	CPSP	CI	0	0	0	0
22	CPSP	CH	0	4	0	6
23	CPSP	CH	0	0	0	2
24	CPSP	CH	0	0	0	4
25	Non-CPSP	CI	0	0	0	0
26	Non-CPSP	CH	0	0	0	0
27	Non-CPSP	CH	0	0	0	0
28	Non-CPSP	CI	0	0	0	0
29	Non-CPSP	CH	0	0	0	0
30	Non-CPSP	CI	0	0	0	0
31	Non-CPSP	CI	0	0	0	0
32	Non-CPSP	CI	0	0	0	0
33	Non-CPSP	CI	0	0	0	0
34	Non-CPSP	CI	0	0	0	0

35	Non-CPSP	CH	0	0	0	0
36	Non-CPSP	CH	0	0	0	0
37	Non-CPSP	CI	0	0	0	0
38	Non-CPSP	CI	0	0	0	0
39	Non-CPSP	CI	0	0	0	0
40	Non-CPSP	CI	0	0	0	0
41	Non-CPSP	CI	0	0	0	0
42	Non-CPSP	CH	0	0	0	0
43	Non-CPSP	CI	0	0	0	0
44	Non-CPSP	CH	0	0	0	0
45	Non-CPSP	CI	0	0	0	0
46	Non-CPSP	CH	0	0	0	0
47	Non-CPSP	CI	0	0	0	0
48	Non-CPSP	CI	0	0	0	0
49	Non-CPSP	CH	0	0	0	8
50	Non-CPSP	CI	0	0	0	0
51	No-pain	CH	0	0	0	0
52	No-pain	CI	0	0	0	0
53	No-pain	CI	0	0	0	0
54	No-pain	CI	0	0	0	0
55	No-pain	CI	0	0	0	0
56	No-pain	CI	0	0	0	0
57	No-pain	CH	0	0	0	0
58	No-pain	CI	0	0	0	0
59	No-pain	CH	0	0	0	0
60	No-pain	CI	0	0	0	0
61	No-pain	CH	0	0	0	0
62	No-pain	CH	0	0	0	0
63	No-pain	CH	0	0	0	0
64	No-pain	CI	0	0	0	0
65	No-pain	CI	0	0	0	0
66	No-pain	CH	0	0	0	0
67	No-pain	CH	0	0	0	0
68	No-pain	CI	0	0	0	0
69	No-pain	CI	0	0	0	0
70	No-pain	CI	0	0	0	0

			Bedside-QST Thermal sensation			
Patients_ID	Groups	Stroke Type	Heat 37˚C Sensation	Heat 45˚C Sensation	Cold 22˚C Sensation	Cold 8˚C Sensation
1	CPSP	CH	0	13	0	16
2	CPSP	CH	0	0	0	0
3	CPSP	CH	8	0	8	8
4	CPSP	CH	15	15	20	18
5	CPSP	CH	0	15	0	3
6	CPSP	CI	2	7	6	8
7	CPSP	CI	9	11	11	11
8	CPSP	CH	20	20	20	20
9	CPSP	CI	15	20	10	10
10	CPSP	CI	8	14	7	8
11	CPSP	CH	10	10	8	6
12	CPSP	CI	2	2	2	1
13	CPSP	CI	5	2	2	4
14	CPSP	CH	3	5	5	5
15	CPSP	CH	0	16	3	18
16	CPSP	CH	0	2	0	2
17	CPSP	CI	5	15	6	18
18	CPSP	CI	7	5	7	7
19	CPSP	CI	2	0	3	0
20	CPSP	CH	0	0	2	0
21	CPSP	CI	0	0	0	0
22	CPSP	CH	7	7	8	8
23	CPSP	CH	0	0	0	0
24	CPSP	CH	8	13	8	15
25	Non-CPSP	CI	12	13	13	14
26	Non-CPSP	CH	7	8	7	8
27	Non-CPSP	CH	11	12	11	12
28	Non-CPSP	CI	12	15	14	15
29	Non-CPSP	CH	10	12	10	12
30	Non-CPSP	CI	14	8	12	9
31	Non-CPSP	CI	11	11	11	11
32	Non-CPSP	CI	10	10	10	10
33	Non-CPSP	CI	10	10	10	0
34	Non-CPSP	CI	4	8	10	10

35	Non-CPSP	CH	0	0	0	5
36	Non-CPSP	CH	10	10	10	10
37	Non-CPSP	CI	10	10	10	10
38	Non-CPSP	CI	7	12	11	11
39	Non-CPSP	CI	8	5	6	5
40	Non-CPSP	CI	17	10	18	10
41	Non-CPSP	CI	10	12	8	10
42	Non-CPSP	CH	10	10	9	10
43	Non-CPSP	CI	5	5	3	10
44	Non-CPSP	CH	8	15	8	15
45	Non-CPSP	CI	10	10	10	10
46	Non-CPSP	CH	15	15	15	11
47	Non-CPSP	CI	7	13	8	13
48	Non-CPSP	CI	9	9	9	9
49	Non-CPSP	CH	2	6	2	8
50	Non-CPSP	CI	5	10	5	11
51	No-pain	CH	10	10	10	10
52	No-pain	CI	10	8	8	9
53	No-pain	CI	10	11	11	11
54	No-pain	CI	10	10	7	10
55	No-pain	CI	10	10	10	10
56	No-pain	CI	1	10	1	10
57	No-pain	CH	10	10	10	10
58	No-pain	CI	10	10	10	10
59	No-pain	CH	12	10	9	10
60	No-pain	CI	8	11	8	10
61	No-pain	CH	9	9	9	9
62	No-pain	CH	10	10	10	10
63	No-pain	CH	1	2	2	2
64	No-pain	CI	10	10	10	10
65	No-pain	CI	10	10	10	10
66	No-pain	CH	8	10	9	6
67	No-pain	CH	8	7	7	7
68	No-pain	CI	9	10	10	10
69	No-pain	CI	10	10	10	10
70	No-pain	CI	10	10	10	10

			Bedside-QST Mechanical pain and sensation					
Patients_ID	Groups	Stroke Type	MDT	MPT	WUR (Pre-Post subtract)	ALL (sum score)	VDT	PPT
1	CPSP	CH	0	0	2	0	0	0
2	CPSP	CH	3	5	1	12	7	3
3	CPSP	CH	15	8	0	0	8	0
4	CPSP	CH	12	4	1	0	8	6
5	CPSP	CH	0	0	0	0	6	0
6	CPSP	CI	16	3	4	0	7	0
7	CPSP	CI	9	9	-1	0	8	0
8	CPSP	CH	9	1	0	5	8	10
9	CPSP	CI	10	0	0	0	8	0
10	CPSP	CI	5	3	2	2	5.5	5
11	CPSP	CH	5	5	3	3	4	10
12	CPSP	CI	14	1	4	6	8	6
13	CPSP	CI	1	0	0	0	8	3
14	CPSP	CH	4	5	0	0	7	4
15	CPSP	CH	0	3	3	7	5.5	0
16	CPSP	CH	2	0	0	0	0	0
17	CPSP	CI	5	3	0	4	7.5	0
18	CPSP	CI	14	0	0	0	7	0
19	CPSP	CI	5	7	1	16	0	8
20	CPSP	CH	0	0	0	0	6.5	2
21	CPSP	CI	7	2	2	0	8	0
22	CPSP	CH	0	0	0	0	8	0
23	CPSP	CH	2	0	0	0	0	0
24	CPSP	CH	6	3	1	2	6	4
25	Non-CPSP	CI	8	0	0	0	7	0
26	Non-CPSP	CH	10	3	2	0	7	4
27	Non-CPSP	CH	10	2	1	0	8	3
28	Non-CPSP	CI	11	4	0	0	8	4
29	Non-CPSP	CH	10	3	1	0	6.5	5
30	Non-CPSP	CI	9	0	0	0	8	1
31	Non-CPSP	CI	10	2	0	0	8	0
32	Non-CPSP	CI	10	0	0	0	5	2
33	Non-CPSP	CI	8	8	1	0	5	0

34	Non-CPSP	CI	10	0	-1	0	5	6
35	Non-CPSP	CH	0	0	0	0	0	0
36	Non-CPSP	CH	10	4	0	0	7	0
37	Non-CPSP	CI	10	0	0	0	7.5	6
38	Non-CPSP	CI	12	2	1	0	6	5
39	Non-CPSP	CI	10	5	3	0	7	7
40	Non-CPSP	CI	8	0	4	0	8	0
41	Non-CPSP	CI	10	0	1	0	8	0
42	Non-CPSP	CH	10	3	0	0	8	4
43	Non-CPSP	CI	5	2	0	0	7	0
44	Non-CPSP	CH	8	0	0	0	8	0
45	Non-CPSP	CI	10	3	4	0	7	0
46	Non-CPSP	CH	12	5	2	0	8	0
47	Non-CPSP	CI	7	0	0	0	6	0
48	Non-CPSP	CI	10	0	0	0	6.5	3
49	Non-CPSP	CH	1	0	0	0	8	5
50	Non-CPSP	CI	1	0	0	0	8	4
51	No-pain	CH	10	0	0	0	8	0
52	No-pain	CI	10	3	0	0	8	0
53	No-pain	CI	9	4	1	0	6.5	0
54	No-pain	CI	10	0	2	0	8	0
55	No-pain	CI	10	3	0	0	8	1
56	No-pain	CI	10	2	0	0	7	4
57	No-pain	CH	10	1	0	0	8	0
58	No-pain	CI	10	1	1	0	8	2
59	No-pain	CH	10	4	1	0	7.5	3
60	No-pain	CI	10	1	1	0	8	0
61	No-pain	CH	10	0	2	0	8	1
62	No-pain	CH	10	3	2	0	7.5	5
63	No-pain	CH	3	2	0	0	0	0
64	No-pain	CI	10	1	0	0	7.5	0
65	No-pain	CI	10	4	0	0	8	0
66	No-pain	CH	10	4	0	0	7.5	8
67	No-pain	CH	9	0	0	0	8	0
68	No-pain	CI	10	0	0	0	7.5	1
69	No-pain	CI	10	2	0	0	8	1
70	No-pain	CI	10	5	0	0	8	0

			Pain interview					
Patients_ID	Groups	Stroke Type	Joint trigger	Subluxation	ROM	MAS	Allodynia	Sensory deficit
1	CPSP	CH	0	0	0	0	1	1
2	CPSP	CH	0	0	0	0	1	1
3	CPSP	CH	0	0	0	0	0	1
4	CPSP	CH	0	0	1	0	0	1
5	CPSP	CH	0	0	0	1	1	1
6	CPSP	CI	1	0	1	0	1	1
7	CPSP	CI	1	0	0	1.5	0	1
8	CPSP	CH	1	1	1	1.5	1	1
9	CPSP	CI	1	0	1	1.5	0	1
10	CPSP	CI	1	1	1	1	1	1
11	CPSP	CH	1	0	1	0	0	1
12	CPSP	CI	0	0	0	0	1	1
13	CPSP	CI	1	0	1	2	0	1
14	CPSP	CH	0	0	1	2	0	1
15	CPSP	CH	0	0	0	1	1	1
16	CPSP	CH	0	0	1	2	1	1
17	CPSP	CI	0	0	0	0	1	1
18	CPSP	CI	0	0	0	0	1	1
19	CPSP	CI	1	1	1	0	1	1
20	CPSP	CH	1	1	1	1	1	1
21	CPSP	CI	0	0	1	0	0	1
22	CPSP	CH	0	1	0	0	1	1
23	CPSP	CH	0	1	0	0	1	1
24	CPSP	CH	1	1	1	1	1	1
25	Non-CPSP	CI	1	0	1	1	0	1
26	Non-CPSP	CH	1	1	1	0	0	0
27	Non-CPSP	CH	0	0	0	0	0	0
28	Non-CPSP	CI	1	1	1	1	0	1
29	Non-CPSP	CH	1	1	1	0	0	1
30	Non-CPSP	CI	1	1	1	0	0	0
31	Non-CPSP	CI	1	0	1	0	0	0
32	Non-CPSP	CI	1	1	1	2	0	1
33	Non-CPSP	CI	0	0	0	0	0	1

34	Non-CPSP	CI	1	1	1	0	0	1
35	Non-CPSP	CH	1	1	1	0	0	1
36	Non-CPSP	CH	1	0	1	0	0	0
37	Non-CPSP	CI	1	0	1	1.5	0	1
38	Non-CPSP	CI	1	0	1	0	0	1
39	Non-CPSP	CI	0	0	1	1	0	0
40	Non-CPSP	CI	1	0	1	0	0	0
41	Non-CPSP	CI	1	0	1	0	0	0
42	Non-CPSP	CH	1	1	1	0	0	0
43	Non-CPSP	CI	1	1	1	0	0	1
44	Non-CPSP	CH	1	0	1	0	0	1
45	Non-CPSP	CI	1	1	1	2	0	0
46	Non-CPSP	CH	1	0	0	0	0	1
47	Non-CPSP	CI	1	0	1	1	0	1
48	Non-CPSP	CI	1	0	1	1	0	0
49	Non-CPSP	CH	1	1	1	1.5	1	1
50	Non-CPSP	CI	1	1	1	1	0	1
51	No-pain	CH	0	0	0	0	0	0
52	No-pain	CI	0	0	0	0	0	0
53	No-pain	CI	0	0	0	0	0	0
54	No-pain	CI	0	0	0	1	0	1
55	No-pain	CI	0	0	0	0	0	0
56	No-pain	CI	0	0	0	0	0	1
57	No-pain	CH	0	0	0	1	0	0
58	No-pain	CI	0	0	0	0	0	0
59	No-pain	CH	0	0	0	0	0	0
60	No-pain	CI	0	0	0	0	0	0
61	No-pain	CH	0	0	1	0	0	0
62	No-pain	CH	0	1	0	0	0	1
63	No-pain	CH	0	1	1	0	0	1
64	No-pain	CI	0	0	0	0	0	0
65	No-pain	CI	0	0	0	0	0	0
66	No-pain	CH	0	0	0	0	0	0
67	No-pain	CH	0	1	0	0	0	1
68	No-pain	CI	0	0	0	0	0	0
69	No-pain	CI	0	0	0	0	0	0
70	No-pain	CI	0	0	0	0	0	0

			physical examination and pain questionnaires				
Patients_ID	Groups	Stroke Type	FMA	NPSI	PDQ	PCS-6	TSK-11
1	CPSP	CH	17.6	31	14	23	28
2	CPSP	CH	74.2	5	10	3	21
3	CPSP	CH	94.1	3	5	11	28
4	CPSP	CH	62.1	6	13	1	17
5	CPSP	CH	90.9	5	10	0	16
6	CPSP	CI	100.0	18	22	20	35
7	CPSP	CI	72.7	28	9	3	14
8	CPSP	CH	77.3	35	20	14	15
9	CPSP	CI	76.5	33	18	8	11
10	CPSP	CI	3.0	15	24	18	33
11	CPSP	CH	80.3	8	7	20	28
12	CPSP	CI	100.0	17	16	6	23
13	CPSP	CI	13.6	25	23	8	27
14	CPSP	CH	66.7	5	9	2	20
15	CPSP	CH	11.8	18	8	10	20
16	CPSP	CH	31.8	0	0	0	11
17	CPSP	CI	31.8	7	9	3	16
18	CPSP	CI	95.5	7	8	10	32
19	CPSP	CI	6.1	28	5	9	18
20	CPSP	CH	6.1	11	4	10	23
21	CPSP	CI	100.0	3	15	6	27
22	CPSP	CH	12.1	6	7	1	14
23	CPSP	CH	60.6	3	13	2	13
24	CPSP	CH	10.6	10	14	9	22
25	Non-CPSP	CI	1.5	3	0	4	17
26	Non-CPSP	CH	6.1	6	6	2	19
27	Non-CPSP	CH	100.0	4	8	2	14
28	Non-CPSP	CI	37.9	4	3	0	20
29	Non-CPSP	CH	75.8	3	3	0	17
30	Non-CPSP	CI	28.8	4	5	4	23
31	Non-CPSP	CI	100.0	0	3	5	25
32	Non-CPSP	CI	0.0	0	7	0	11
33	Non-CPSP	CI	94.1	2	3	3	15
34	Non-CPSP	CI	19.7	0	1	3	16
35	Non-CPSP	CH	43.9	10	11	5	11

36	Non-CPSP	CH	74.2	0	3	7	12
37	Non-CPSP	CI	42.4	17	14	0	25
38	Non-CPSP	CI	93.9	11	4	11	24
39	Non-CPSP	CI	21.2	3	3	8	22
40	Non-CPSP	CI	60.6	15	4	13	18
41	Non-CPSP	CI	100.0	6	0	9	23
42	Non-CPSP	CH	18.2	0	1	6	16
43	Non-CPSP	CI	39.4	2	4	11	25
44	Non-CPSP	CH	66.7	0	9	21	41
45	Non-CPSP	CI	7.6	0	1	0	11
46	Non-CPSP	CH	87.9	0	4	6	21
47	Non-CPSP	CI	8.8	12	13	24	39
48	Non-CPSP	CI	77.3	4	2	1	16
49	Non-CPSP	CH	30.3	16	10	19	24
50	Non-CPSP	CI	10.6	25	10	6	24
51	No-pain	CH	100.0	0	0	0	11
52	No-pain	CI	100.0	0	0	0	11
53	No-pain	CI	100.0	0	0	0	11
54	No-pain	CI	92.4	0	0	0	11
55	No-pain	CI	93.9	0	0	0	11
56	No-pain	CI	19.7	0	0	0	11
57	No-pain	CH	100.0	0	0	0	11
58	No-pain	CI	93.9	0	0	0	11
59	No-pain	CH	100.0	0	0	0	11
60	No-pain	CI	95.5	0	0	0	11
61	No-pain	CH	97.0	0	0	0	11
62	No-pain	CH	72.7	0	0	0	11
63	No-pain	CH	40.9	0	0	0	11
64	No-pain	CI	100.0	0	0	0	11
65	No-pain	CI	100.0	0	0	0	11
66	No-pain	CH	100.0	0	0	0	11
67	No-pain	CH	16.7	0	0	0	11
68	No-pain	CI	100.0	0	0	0	11
69	No-pain	CI	98.5	0	0	0	11
70	No-pain	CI	100.0	0	0	0	11

The table summarizes the data outlining the individual patient's profile. CPSP, central post-stroke pain; non-CPSP, non-central post-stroke pain; CI, cerebral infarction; CH, cerebral hemorrhage.

Supplementary Figure 1

Sample Size (used G*power)

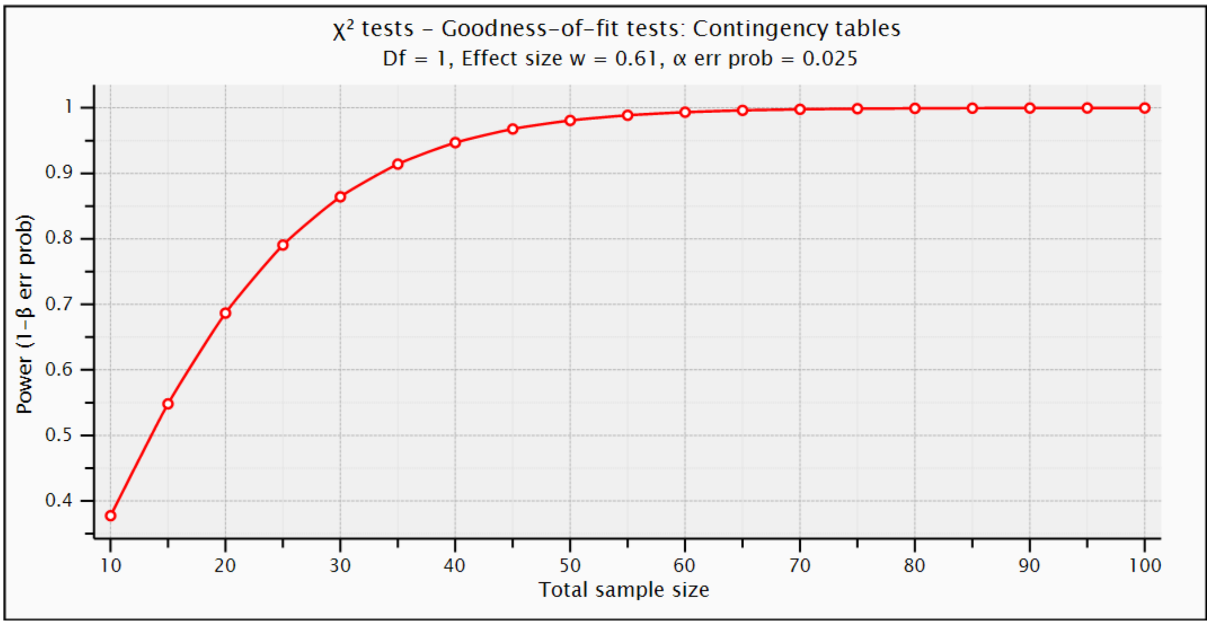

The validity of the sample size was set based on the with/without of abnormal sensation in the CPSP and non-CPSP based on a previous study.[1] We calculated the adequate sample size considering the impact of multiplicity (Df = 1, effect size = 0.61, α error probability = 0.025). The power of sample size (y-axis) is 0.98 with a total sample of 50 (x-axis).

Supplementary reference (Supplementary Figure 1)

1. Barbosa LM, Da Silva VA, De Lima Rodrigues AL, et al. Dissecting central post-stroke pain: A controlled symptom-psychophysical characterization. *Brain Commun*. 2022;4(3). doi:10.1093/braincomms/fcac090

Supplementary Table 2

Comparison of outcomes in CH and CI

				Significance tests
The difference in the outcomes between CH group and CI group	CH (N = 31)	CI (N = 39)	Detection ratio (%)	CI vs CH (p-value)
Bedside-QST				
Heat 37˚C allodynia	2.0	0.0	6% vs 0%	0.19
Heat 37˚C Hypersensitivity	3.0	3.0	9.6% vs 7.7%	1
Heat 37˚C Hypoesthesia	13.0	13.0	41.9% vs 33.3%	0.62
Heat 45˚C Hyperalgesia	2.0	3.0	6% vs 8%	1
Heat 45˚C Hypersensitivity	3.0	3.0	9.6% vs 7.7%	1
Heat 45˚C Hypoesthesia	13.0	13.0	42% vs 33%	0.62
Cold 22˚C allodynia	6.0	3.0	19% vs 8%	0.17
Cold 22˚C Hypersensitivity	8.0	6.0	25.8% vs 15.4%	0.37
Cold 22˚C Hypoesthesia	11.0	8.0	35.5% vs 20.5%	0.19
Cold 8˚C Hyperalgesia	10.0	3.0	32% vs 8%	< 0.05*
Cold 8˚C Hypersensitivity	6.0	4.0	19.4% vs 10.3%	0.32
Cold 8˚C Hypoesthesia	11.0	7.0	35.5% vs 17.9%	0.11
MDT Hypersensitivity	3.0	5.0	9.7% vs 12.8%	1
MDT Hypoesthesia	17.0	14.0	54% vs 35.9%	0.15
MPT Hypersensitivity	9.0	8.0	29% vs 20.5%	0.57
MPT Hypoesthesia	12.0	15.0	38.7% vs 38.4%	1
WUR	7.0	8.0	22.6% vs 20.5%	1
DMA	5.0	4.0	16.1% vs 10.3%	0.49
VDT Hypoesthesia	6.0	1.0	19.4% vs 2.6%	< 0.05*
PPT Hypersensitivity	7.0	7.0	22.5% vs 17.9%	0.76
PPT Hypoesthesia	15.0	21.0	48.4% vs 53.8%	0.81
Physical and Pain-related factor				
FMA	58.9 ± 32.8	62.5 ± 38.8	-	0.91
NPSI	5.9 ± 8.5	7.4 ± 9.6	-	0.44
PDQ	6.1 ± 5.4	5.7 ± 7.1	-	0.73
PCS-6	5.6 ± 7.0	4.9 ± 6.0	-	0.8
TSK-11	17.4 ± 7.0	18.5 ± 7.8	-	0.7
Trigger Joint Pain	12.0	21.0	38.7% vs 53.8%	0.23
Sabluxation	13.0	9.0	41.9% vs 23.1%	0.12

ROM	16.0	22.0	51.6% vs 56.4%	0.81
MAS	9.0	14.0	29.0% vs 35.9%	0.61
Allodynia	11.0	6.0	35.5% vs 15.4%	0.09
Sensory Disturbance	22	22	71% vs 56.4%	0.22

The table indicates outcomes (bedside-QST, physical factor, and pain-related factors) in CH and CI. *P < 0.05, the Willcoxon test, and Fisher's exact tests. CI, cerebral infarction; CH, cerebral hemorrhage; QST, Quantitative sensory testing; MDT, mechanical detection threshold; MPT, mechanical pain threshold; WUR, wind-up; DMA, dynamic mechanical allodynia; VDT, vibration detection threshold; PPT, pressure pain threshold; FMA, Fugl-Meyer assessment; NPSI, Neuropathic pain symptom inventory; PDQ, pain detection questionnaire; PCS, pain catastrophizing scale; TSK-11, Tampa scale for kinesiophobia-11; ROM, range of motion; MAS, modified ashworth scale.

Supplementary Figure 2

Characteristics of pain and pain quality in CPSP and non-CPSP patient

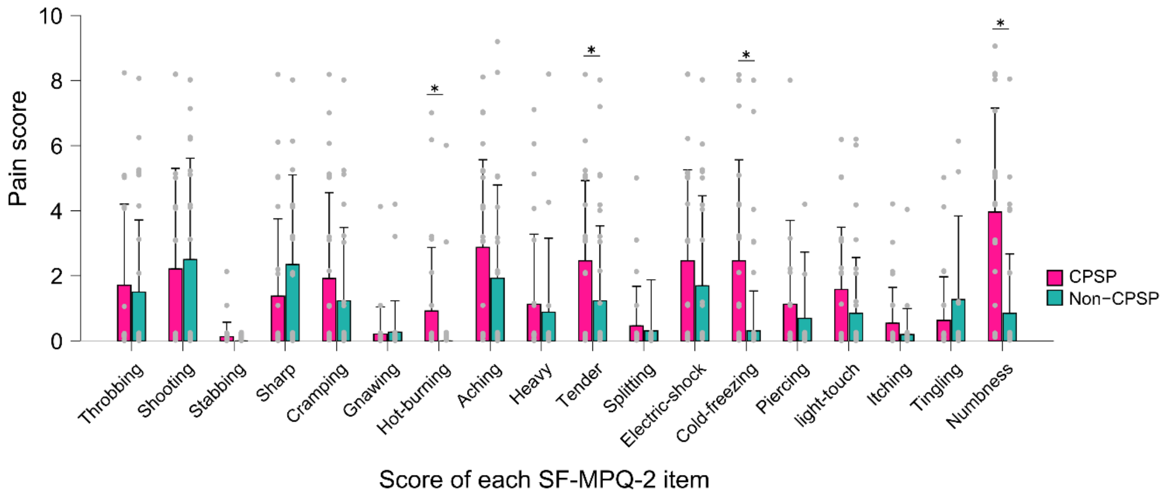

The bar and rhombus graphs show pain and pain quality characteristics in CPSP (pink) and non-CPSP (green) patients. Pain quality was assessed using the sensory items in the Short Form-McGill Pain Questionnaire-2 (SF-MPQ-2) (x-axis). Pain scores are shown as the means ± SD (y-axis). *Kruskal–Wallis test with Bonferroni–Holm post hoc correction for multiple comparisons, $P < 0.05$. Individual gray dots represent the pain scores for each patient.

Supplementary Table 3

CPSP model variable selected for Akaike's information criteria (AIC)

CPSP model

Model number	Variables (Intercept)	cold22 low	cold22 pain	cold8low	cold8 pain	Heat37 low	heat45 low	NPSI	df	logLik	AIC	delta	weight
		Beta											
79	20.57	NA	-18.94	-2.98	-2.61	NA	NA	0.17	5	-17.24	44.47	0.00	0.25
80	20.83	0.75	-19.09	-3.52	-2.97	NA	NA	0.18	6	-17.06	46.12	1.65	0.11
111	20.63	NA	-18.99	-3.13	-2.70	NA	0.25	0.18	6	-17.21	46.42	1.94	0.09
95	20.63	NA	-18.97	-2.92	-2.56	-0.16	NA	0.17	6	-17.22	46.44	1.97	0.09
96	21.34	1.32	-19.39	-3.68	-3.02	-0.73	NA	0.18	7	-16.86	47.73	3.25	0.05
71	19.87	NA	-20.48	-2.82	NA	NA	NA	0.18	4	-19.96	47.93	3.45	0.04
112	21.19	1.45	-19.36	-3.62	-3.06	NA	-0.72	0.18	7	-16.97	47.95	3.48	0.04
77	2.73	NA	NA	-2.54	-3.72	NA	NA	0.17	4	-20.01	48.03	3.55	0.04
127	21.04	NA	-19.29	-3.21	-2.67	-0.64	0.76	0.18	7	-17.09	48.18	3.71	0.04
87	20.22	NA	-20.47	-2.62	NA	-0.77	NA	0.17	5	-19.53	49.06	4.59	0.03
72	18.69	-0.68	-19.11	-2.38	NA	NA	NA	0.17	5	-19.74	49.48	5.01	0.02
103	20.07	NA	-20.52	-2.53	NA	NA	-0.57	0.17	5	-19.77	49.54	5.07	0.02
128	21.38	1.45	-19.43	-3.70	-3.04	-0.66	-0.19	0.18	8	-16.86	49.72	5.24	0.02
78	2.83	0.31	NA	-2.74	-3.92	NA	NA	0.17	5	-19.98	49.96	5.49	0.02
109	2.77	NA	NA	-2.46	-3.72	NA	-0.13	0.17	5	-20.00	50.01	5.53	0.02
93	2.73	NA	NA	-2.55	-3.73	0.04	NA	0.17	5	-20.01	50.03	5.55	0.02
88	20.11	-0.18	-20.35	-2.53	NA	-0.69	NA	0.17	6	-19.52	51.04	6.57	0.01
119	20.22	NA	-20.47	-2.65	NA	-0.82	0.08	0.17	6	-19.53	51.06	6.59	0.01
76	18.42	-1.83	-17.60	NA	-2.02	NA	NA	0.14	5	-20.64	51.28	6.81	0.01
104	18.79	-0.49	-19.21	-2.39	NA	NA	-0.21	0.17	6	-19.73	51.47	6.99	0.01
110	3.05	0.59	NA	-2.71	-4.09	NA	-0.39	0.17	6	-19.92	51.85	7.37	0.01
94	2.86	0.35	NA	-2.74	-3.92	-0.08	NA	0.17	6	-19.98	51.96	7.48	0.01
125	2.76	NA	NA	-2.47	-3.76	0.15	-0.23	0.17	6	-19.99	51.99	7.51	0.01
68	18.18	-2.19	-18.90	NA	NA	NA	NA	0.14	4	-22.00	52.00	7.52	0.01
107	17.98	NA	-17.26	NA	-2.31	NA	-1.43	0.14	5	-21.34	52.68	8.21	0.00
120	19.99	-0.46	-20.21	-2.53	NA	-0.81	0.41	0.17	7	-19.50	52.99	8.52	0.00
92	18.45	-1.70	-17.59	NA	-2.01	-0.21	NA	0.14	6	-20.62	53.24	8.76	0.00
108	18.37	-1.72	-17.54	NA	-2.03	NA	-0.13	0.14	6	-20.64	53.27	8.80	0.00

74	1.85	-1.61	NA	NA	-3.14	NA	NA	0.15	4	-22.84	53.68	9.21	0.00
91	18.18	NA	-17.50	NA	-2.40	-1.16	NA	0.13	5	-21.88	53.76	9.29	0.00
126	3.03	0.57	NA	-2.70	-4.10	0.08	-0.43	0.17	7	-19.92	53.84	9.37	0.00
84	18.15	-2.00	-18.80	NA	NA	-0.30	NA	0.14	5	-21.95	53.90	9.43	0.00
75	17.57	NA	-17.21	NA	-2.82	NA	NA	0.14	4	-22.96	53.91	9.44	0.00
100	18.19	-2.19	-18.91	NA	NA	NA	0.00	0.14	5	-22.00	54.00	9.52	0.00
105	1.98	NA	NA	NA	-3.55	NA	-1.41	0.14	4	-23.01	54.02	9.55	0.00
99	18.00	NA	-19.15	NA	NA	NA	-1.72	0.14	4	-23.27	54.54	10.07	0.00
123	18.01	NA	-17.27	NA	-2.29	-0.20	-1.28	0.14	6	-21.33	54.65	10.18	0.00
106	1.95	-1.05	NA	NA	-3.16	NA	-0.81	0.15	5	-22.52	55.05	10.57	0.00
124	18.48	-1.73	-17.63	NA	-2.01	-0.23	0.06	0.14	7	-20.62	55.24	10.76	0.00
73	1.36	NA	NA	NA	-3.81	NA	NA	0.15	3	-24.73	55.45	10.98	0.00
90	1.86	-1.51	NA	NA	-3.10	-0.19	NA	0.15	5	-22.82	55.64	11.17	0.00
116	18.27	-2.21	-18.91	NA	NA	-0.41	0.32	0.14	6	-21.93	55.87	11.39	0.00
121	1.98	NA	NA	NA	-3.57	0.05	-1.44	0.14	5	-23.01	56.02	11.55	0.00
89	1.64	NA	NA	NA	-3.46	-0.91	NA	0.14	4	-24.03	56.05	11.58	0.00
83	17.77	NA	-19.01	NA	NA	-1.46	NA	0.13	4	-24.03	56.05	11.58	0.00
115	18.02	NA	-19.09	NA	NA	-0.39	-1.43	0.14	5	-23.21	56.42	11.95	0.00
122	1.94	-1.09	NA	NA	-3.20	0.23	-0.93	0.15	6	-22.50	57.00	12.53	0.00
67	17.04	NA	-19.20	NA	NA	NA	NA	0.14	3	-26.04	58.07	13.60	0.00
15	21.24	NA	-18.29	-2.11	-2.92	NA	NA	NA	4	-25.92	59.83	15.36	0.00
70	0.14	-1.38	NA	-1.70	NA	NA	NA	0.17	4	-26.34	60.69	16.21	0.00
31	21.37	NA	-18.41	-1.84	-2.61	-0.77	NA	NA	5	-25.43	60.85	16.38	0.00
69	-0.26	NA	NA	-2.48	NA	NA	NA	0.18	3	-27.66	61.32	16.85	0.00
85	0.15	NA	NA	-2.16	NA	-0.97	NA	0.16	4	-26.76	61.53	17.05	0.00
47	20.97	NA	-18.08	-1.90	-2.76	NA	-0.36	NA	5	-25.83	61.67	17.19	0.00
16	21.24	-0.02	-18.29	-2.10	-2.91	NA	NA	NA	5	-25.92	61.83	17.36	0.00
86	0.29	-1.09	NA	-1.67	NA	-0.56	NA	0.17	5	-26.11	62.22	17.75	0.00
66	-0.25	-2.33	NA	NA	NA	NA	NA	0.16	3	-28.15	62.31	17.83	0.00
32	21.69	0.88	-18.45	-2.36	-2.98	-1.11	NA	NA	6	-25.21	62.41	17.94	0.00
101	-0.05	NA	NA	-2.06	NA	NA	-0.75	0.16	4	-27.22	62.44	17.96	0.00
102	0.15	-1.31	NA	-1.66	NA	NA	-0.13	0.17	5	-26.33	62.67	18.19	0.00
63	21.81	NA	-18.76	-2.02	-2.71	-1.08	0.50	NA	6	-25.34	62.69	18.21	0.00
48	20.93	0.77	-17.88	-2.20	-3.00	NA	-0.81	NA	6	-25.73	63.45	18.98	0.00
117	0.16	NA	NA	-2.10	NA	-0.88	-0.16	0.16	5	-26.75	63.50	19.03	0.00
82	-0.07	-1.98	NA	NA	NA	-0.64	NA	0.15	4	-27.83	63.65	19.18	0.00
12	20.17	-1.63	-18.34	NA	-2.02	NA	NA	NA	4	-27.83	63.65	19.18	0.00
98	-0.13	-1.92	NA	NA	NA	NA	-0.64	0.15	4	-27.87	63.74	19.26	0.00

23	20.09	NA	-19.36	-1.62	NA	-1.20	NA	NA	4	-27.88	63.77	19.29	0.00
13	3.88	NA	NA	-1.94	-3.84	NA	NA	NA	3	-29.00	63.99	19.52	0.00
118	0.29	-1.16	NA	-1.74	NA	-0.65	0.22	0.17	6	-26.09	64.18	19.71	0.00
64	21.68	0.88	-18.44	-2.36	-2.98	-1.11	0.01	NA	7	-25.21	64.41	19.94	0.00
43	19.36	NA	-17.62	NA	-2.27	NA	-1.37	NA	4	-28.25	64.50	20.02	0.00
27	20.00	NA	-18.25	NA	-2.27	-1.37	NA	NA	4	-28.25	64.50	20.02	0.00
7	19.90	NA	-19.62	-2.03	NA	NA	NA	NA	3	-29.34	64.68	20.21	0.00
4	19.72	-2.03	-19.43	NA	NA	NA	NA	NA	3	-29.34	64.68	20.21	0.00
28	20.26	-1.15	-18.33	NA	-1.98	-0.75	NA	NA	5	-27.47	64.94	20.47	0.00
8	19.99	-1.20	-19.45	-1.20	NA	NA	NA	NA	4	-28.56	65.11	20.64	0.00
45	3.84	NA	NA	-1.53	-3.62	NA	-0.73	NA	4	-28.58	65.16	20.68	0.00
39	19.87	NA	-19.37	-1.49	NA	NA	-0.96	NA	4	-28.59	65.18	20.70	0.00
29	3.86	NA	NA	-1.70	-3.55	-0.65	NA	NA	4	-28.62	65.24	20.77	0.00
114	-0.05	-1.82	NA	NA	NA	-0.46	-0.40	0.15	5	-27.74	65.48	21.01	0.00
44	19.91	-1.20	-18.06	NA	-2.04	NA	-0.49	NA	5	-27.75	65.50	21.03	0.00
24	20.05	-0.36	-19.29	-1.42	NA	-1.05	NA	NA	5	-27.83	65.67	21.19	0.00
20	19.60	-1.48	-19.19	NA	NA	-0.83	NA	NA	4	-28.87	65.74	21.26	0.00
55	20.09	NA	-19.36	-1.62	NA	-1.21	0.00	NA	5	-27.88	65.77	21.29	0.00
59	19.64	NA	-17.83	NA	-2.20	-0.78	-0.78	NA	5	-27.96	65.93	21.45	0.00
14	3.91	0.13	NA	-2.03	-3.90	NA	NA	NA	4	-28.99	65.97	21.50	0.00
11	19.39	NA	-18.00	NA	-2.80	NA	NA	NA	3	-30.25	66.49	22.02	0.00
36	19.57	-1.67	-19.28	NA	NA	NA	-0.41	NA	4	-29.29	66.58	22.10	0.00
41	3.01	NA	NA	NA	-3.40	NA	-1.47	NA	3	-30.31	66.62	22.14	0.00
46	4.15	0.90	NA	-1.99	-4.08	NA	-1.07	NA	5	-28.32	66.64	22.17	0.00
97	-0.65	NA	NA	NA	NA	NA	-1.77	0.13	3	-30.35	66.70	22.23	0.00
19	19.12	NA	-19.23	NA	NA	-1.69	NA	NA	3	-30.37	66.74	22.26	0.00
35	19.12	NA	-19.23	NA	NA	NA	-1.69	NA	3	-30.37	66.74	22.26	0.00
30	4.04	0.65	NA	-2.09	-3.83	-0.84	NA	NA	5	-28.47	66.93	22.46	0.00
40	19.84	-0.71	-19.29	-1.24	NA	NA	-0.53	NA	5	-28.47	66.93	22.46	0.00
60	20.34	-1.21	-18.41	NA	-1.97	-0.79	0.10	NA	6	-27.47	66.94	22.46	0.00
61	3.84	NA	NA	-1.54	-3.54	-0.35	-0.48	NA	5	-28.52	67.03	22.56	0.00
56	20.17	-0.57	-19.39	-1.42	NA	-1.18	0.34	NA	6	-27.80	67.61	23.13	0.00
81	-0.68	NA	NA	NA	NA	-1.66	NA	0.13	3	-30.83	67.66	23.19	0.00
52	19.77	-1.66	-19.35	NA	NA	-0.95	0.30	NA	5	-28.85	67.69	23.22	0.00
51	19.15	NA	-19.11	NA	NA	-0.96	-0.96	NA	4	-29.88	67.77	23.29	0.00
113	-0.48	NA	NA	NA	NA	-0.84	-1.20	0.13	4	-29.89	67.78	23.31	0.00
10	3.10	-1.40	NA	NA	-3.35	NA	NA	NA	3	-30.98	67.97	23.49	0.00
42	3.06	-0.55	NA	NA	-3.23	NA	-1.15	NA	4	-30.13	68.27	23.79	0.00

25	2.77	NA	NA	NA	-3.27	-1.22	NA	NA	3	-31.17	68.35	23.87	0.00
62	4.19	1.00	NA	-2.04	-4.02	-0.46	-0.79	NA	6	-28.21	68.41	23.94	0.00
57	3.01	NA	NA	NA	-3.32	-0.34	-1.24	NA	4	-30.24	68.49	24.01	0.00
26	3.04	-0.96	NA	NA	-3.11	-0.77	NA	NA	4	-30.53	69.06	24.58	0.00
9	2.48	NA	NA	NA	-3.81	NA	NA	NA	2	-32.86	69.72	25.25	0.00
58	3.06	-0.51	NA	NA	-3.18	-0.27	-0.99	NA	5	-30.09	70.19	25.71	0.00
3	18.57	NA	-19.69	NA	NA	NA	NA	NA	2	-34.03	72.05	27.58	0.00
65	-1.73	NA	NA	NA	NA	NA	NA	0.15	2	-34.29	72.58	28.11	0.00
21	1.19	NA	NA	-1.33	NA	-1.57	NA	NA	3	-36.10	78.21	33.74	0.00
18	1.01	-1.31	NA	NA	NA	-1.33	NA	NA	3	-36.46	78.91	34.44	0.00
22	1.29	-0.73	NA	-0.96	NA	-1.32	NA	NA	4	-35.75	79.49	35.02	0.00
34	0.93	-1.23	NA	NA	NA	NA	-1.28	NA	3	-36.84	79.67	35.20	0.00
53	1.18	NA	NA	-1.15	NA	-1.20	-0.59	NA	4	-35.89	79.77	35.30	0.00
37	0.97	NA	NA	-1.10	NA	NA	-1.47	NA	3	-36.95	79.89	35.42	0.00
49	0.66	NA	NA	NA	NA	-1.15	-1.15	NA	3	-37.16	80.32	35.85	0.00
50	1.02	-1.08	NA	NA	NA	-0.96	-0.67	NA	4	-36.17	80.34	35.86	0.00
17	0.47	NA	NA	NA	NA	-1.97	NA	NA	2	-38.19	80.37	35.90	0.00
33	0.47	NA	NA	NA	NA	NA	-1.97	NA	2	-38.19	80.37	35.90	0.00
2	0.77	-2.06	NA	NA	NA	NA	NA	NA	2	-38.44	80.88	36.41	0.00
38	1.12	-0.87	NA	-0.72	NA	NA	-1.14	NA	4	-36.45	80.90	36.43	0.00
54	1.27	-0.63	NA	-0.87	NA	-1.09	-0.43	NA	5	-35.64	81.28	36.80	0.00
6	1.06	-1.47	NA	-0.98	NA	NA	NA	NA	3	-37.65	81.31	36.83	0.00
5	0.69	NA	NA	-1.90	NA	NA	NA	NA	2	-39.55	83.10	38.62	0.00
1	-0.65	NA	NA	NA	NA	NA	NA	NA	1	-45.00	92.01	47.53	0.00

The model fitting was evaluated stepwise using AIC to examine the best relationships from significant variables. The top line represents the selected variables in the optimal model fitting. AIC, akaike’s information criterion; NPSI, neuropathic pain symptoms inventory.

Supplementary Table 4

Non-CPSP model variable selected for Akaike's information criteria (AIC)

Non-CPSP model

Model number	Variables (Intercept)	FMA	NPSI	ROM	tri	df	logLik	AIC	delta	weight
		Beta								
11	-2.11	NA	-0.17	NA	5.01	3	-23.27	52.53	0.00	0.50
15	-2.31	NA	-0.17	0.83	4.41	4	-23.03	54.07	1.53	0.23
12	-1.89	0.00	-0.17	NA	4.92	4	-23.24	54.48	1.94	0.19
16	-2.26	0.00	-0.17	0.81	4.40	5	-23.03	56.06	3.53	0.08
9	-2.43	NA	NA	NA	3.26	2	-30.65	65.31	12.78	0.00
13	-2.65	NA	NA	0.87	2.67	3	-30.28	66.56	14.03	0.00
10	-2.37	0.00	NA	NA	3.24	3	-30.65	67.30	14.77	0.00
14	-2.76	0.00	NA	0.91	2.69	4	-30.27	68.54	16.01	0.00
7	-2.05	NA	-0.08	3.20	NA	3	-32.84	71.69	19.15	0.00
8	-1.56	-0.01	-0.08	3.01	NA	4	-32.65	73.29	20.76	0.00
5	-2.27	NA	NA	2.70	NA	2	-35.45	74.89	22.36	0.00
6	-2.03	0.00	NA	2.60	NA	3	-35.38	76.77	24.23	0.00
4	0.94	-0.02	-0.05	NA	NA	3	-42.35	90.70	38.16	0.00
2	0.39	-0.02	NA	NA	NA	2	-43.57	91.13	38.60	0.00
1	-0.53	NA	NA	NA	NA	1	-46.18	94.36	41.83	0.00
3	-0.38	NA	-0.02	NA	NA	2	-45.86	95.73	43.19	0.00

The model fitting was evaluated stepwise using AIC to examine the best relationships from significant variables. The top line represents the selected variables in the optimal model fitting. AIC, akaike's information criterion; NPSI, neuropathic pain symptoms inventory; FMA, Fugl-Meyer assessment; ROM, range of motion.

Supplementary Table 5

Statistical ratio and brain areas in three groups (CPSP, non-CPSP and no-pain)

Significant Brain Lesion area in allodynia with Cold stimulation22˚C			
VLSM	FDR (>0.05)	Z=2.231	
	Permutation FWE (>0.05)	Z=3.373	
	Area	XYZ	Atlas
	Putamen	29×-17.8×5.7	ALL
	Retrolenticular part of internal capsule	28.2×-21.5×4.2	JHU
	External capsule	28.9×-19.3×8.6	JHU
VDSM	FDR (>0.05)	n.s	
	Permutation FWE (>0.05)	n.s	

Significant Brain Lesion area in hyperalgesia with Cold stimulation 8˚C			
VLSM	FDR (>0.05)	Z=1.97	
	Permutation FWE (>0.05)	Z=3.56	
	Area	XYZ	Atlas
	Putamen	29×-17.8×5.7	ALL
	Insula	28.9×-19.3×18.9	ALL
	Retrolenticular part of internal capsule	27.4×-20.7×5.6	JHU
	External capsule	26.7×-20.8×5.6	JHU
VDSM	FDR (>0.05)	Z=2.29	
	Permutation FWE (>0.05)	Z=3.97	
	Tract (HCP 1065 atlas)		
	Cingulum Frontal Parahippocampal		

Significant Brain Lesion area in Hypoesthesia with Heat stimulation 37˚C			
VLSM	FDR (>0.05)	Z=2.23	
	Permutation FWE (>0.05)	Z=3.501	
	Area	XYZ	Atlas
	Insula	34.8×-13.4×4.9	ALL
VDSM	FDR (>0.05)	Z=2.58	
	Permutation FWE (>0.05)	Z=4.05	
	Tract (HCP 1065 atlas)		
	Superior longitudinal fasciculus		

Significant Brain Lesion area in Hypoesthesia with Heat stimulation 45˚C			
VLSM	FDR (>0.05)	Z=1.79	
	Permutation FWE (>0.05)	Z=3.501	
	Area	XYZ	Atlas
	Insula	34.4×-13.4×4.9	ALL
	Putamen	31.9×-11.9×4.9	ALL
	Rolandic Oper	37.1×-20.1×24.1	ALL
	Hippocampus	34.1×-18.6×-7.6	ALL
	Posterior corona radiata	28.2×-25.2×24.1	JHU
	Superior longitudinal fasciculus	31.9×-9.7×21.9	JHU
	External capsule	31.9×-11.9×-4.9	JHU
	Sagittal stratum (include inferior longitidinal fasciculus and inferior fronto occipital fasciculus)	34.1×-18.6×-7.6	JHU
VDSM	FDR (>0.05)	Z=1.77	
	Permutation FWE (>0.05)	Z=4.08	
	Tract (HCP 1065 atlas)		
	Corpus Callosum		
	Reticulospinal tract		

Significant Brain Lesion area in Hypoesthesia with Touch			
VLSM	FDR (>0.05)	Z=2.69	
	Permutation FWE (>0.05)	Z=3.479	
	Area	XYZ	Atlas
	Hippocampus	34.9×-20.1×-6.1	ALL
	Sagittal stratum (include inferior longitidinal fasciculus and inferior fronto occipital fasciculus)	34.9×-20.1×-6.1	JHU
VDSM	FDR (>0.05)	n.s	
	Permutation FWE (>0.05)	n.s	

Significant Brain Lesion area in Hypoesthesia with VDT			
VLSM	FDR (>0.05)	Z=2.05	
	Permutation FWE (>0.05)	Z=3.195	
	Area	XYZ	Atlas
	Superior longitudinal fasciculus	29.7×-2.3×-18.9	JHU
VDSM	FDR (>0.05)	n.s	
	Permutation FWE (>0.05)	n.s	

Each table presents the statistical ratio (Z ratio) and significantly detected areas in VLSM and VDSM. The volume of regions of VLSM presents x,y, and z. VLSM and VDSM used the JHU atlas, the AAL atlas, and the HCP 1065 atlas respectively. FDR, P<0.05, permutation family-wise error, P<0.05. FDR, false discovery rate; FWE, family-wise error; VLSM, voxel-based lesion-symptom mapping; VDSM, voxel-based disconnection-symptom mapping; JHU, the Johns Hopkins University white-matter tractography atlas; AAL, the Automated Anatomical Labelling atlas; HCP, Human Connectome Project atlas; VDT, vibration detection threshold; CPSP, central post-stroke pain; non-CPSP, non-central post-stroke pain.

Supplementary Figure 3

The results of VLSM in CPSP and no-pain groups

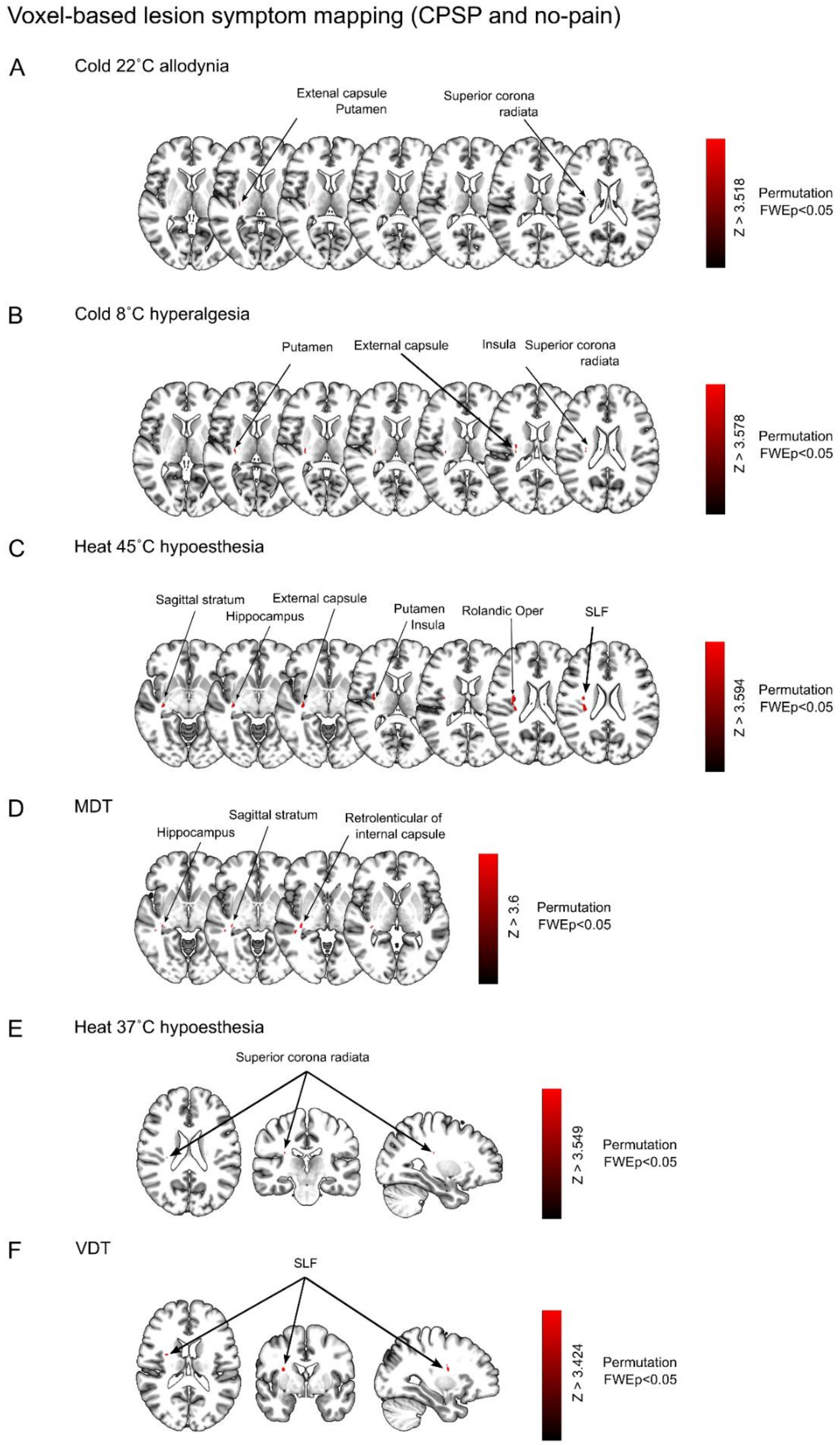

(**A**–**F**) The VLSM results indicate the areas associated with significant items in the bedside-QST among the no-pain and CPSP groups (only voxels involving at least 9 patients were included in VLSM). The color bars represent *Z*-scores; color-highlighted voxels are expressed at above-threshold regions (cold allodynia 22°C: $Z = 3.518$; cold hyperalgesia 8°C: $Z = 3.578$; heat hypoesthesia at 45°C: $Z = 3.594$; MDT: $Z = 3.6$; heat hypoesthesia at 37°C: $Z = 3.549$; VDT: $Z = 3.424$). The lesion map in the VLSM presents only the right side (false-discovery rate, $P < 0.05$, permutation family-wise error, $P < 0.05$). CPSP, central post-stroke pain; SLF, superior longitudinal fasciculus; FDR, false discovery rate; FWE, family-wise error; VLSM, voxel-based lesion–symptom mapping.

Supplementary Figure 6

The results of VDSM in CPSP and no-pain groups

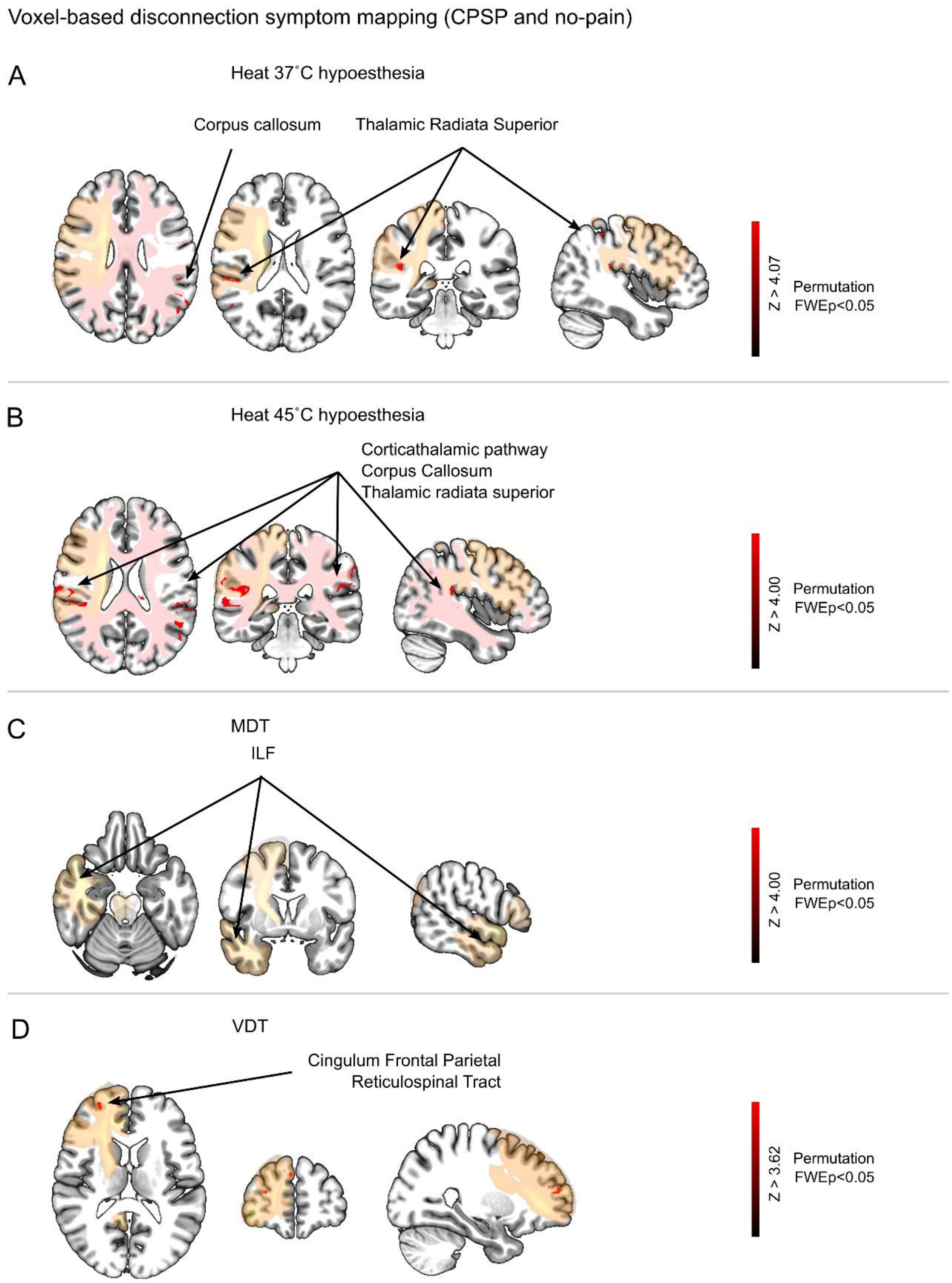

(**A**–**D**) The VDSM results indicate the disconnection of white matter associated with significant items in the bedside-QST among the no-pain and CPSP groups (20% of the sample). The color bars represent *Z*-scores; color-highlighted disconnection is shown at above-threshold regions (heat hypoesthesia at 37°C: $Z = 4.07$; heat hypoesthesia at 45°C: $Z = 4.0$; hypoesthesia at MDT: $Z = 4.0$; hypoesthesia at VDT: $Z = 3.62$). The disconnection map in the VDSM presents both intrahemispheric and interhemispheric (false-discovery rate, $P < 0.05$, permutation family-wise error, $P < 0.05$). CPSP, central post-stroke pain; FDR, false discovery rate; FWE, family-wise error; ILF, inferior longitudinal fasciculus; VDSM, voxel-based disconnection–symptom mapping.

Supplementary Table 6

Statistical ratio and brain areas in the CPSP and no-pain groups.

Significant Brain Lesion area in allodynia with Cold stimulation22˚C			
VLSM	FDR (>0.05)	Z=2.79	
	Permutation FWE (>0.05)	Z=3.518	
	Area	XYZ	Atlas
	Putamen	29×-19.3×7.8	ALL
	Superior corona radiata	28.2×-11.9×18.9	JHU
	External capsule	29×-19.3×7.8	JHU
VDSM	FDR (>0.05)	n.s	
	Permutation FWE (>0.05)	n.s	

Significant Brain Lesion area in hyperalgesia with Cold stimulation 8˚C			
VLSM	FDR (>0.05)	Z=2.47	
	Permutation FWE (>0.05)	Z=3.578	
	Area	XYZ	Atlas
	Putamen	29×-20×8.6	ALL
	Insula	28.9×-19.3×20.4	ALL
	Superior corona radiata	28.9×-17.8×20.4	JHU
	External capsule	28.2×-12.6×15.9	JHU
VDSM	FDR (>0.05)	n.s	
	Permutation FWE (>0.05)	n.s	

Significant Brain Lesion area in Hypoesthesia with Heat stimulation 37˚C			
VLSM	FDR (>0.05)	Z=2.41	
	Permutation FWE (>0.05)	Z=3.549	
	Area	XYZ	Atlas
	Superior corona radiata	28.9×-23.0×21.2	JHU
VDSM	FDR (>0.05)	Z=2.22	
	Permutation FWE (>0.05)	Z=4.07	
	Tract (HCP 1065 atlas)		
	Thalamic Radiata Superior		
	Corpus Callosum		

Significant Brain Lesion area in Hypoesthesia with Heat stimulation 45˚C			
VLSM	FDR (>0.05)	Z=1.99	
	Permutation FWE (>0.05)	Z=3.594	
	Area	XYZ	Atlas
	Insula	35.6×-11.9×4.9	ALL
	Putamen	33.3×-11.2×4.2	ALL
	Rolandic Oper	34.8×-16.4×20.4	ALL
	Hippocampus	34.1×-18.6×-6.1	ALL
	Superior longitudinal fasciculus	32.6×-8.9×21.9	JHU
	External capsule	33.3×-8.2×-4.9	JHU
	Sagittal stratum (include inferior longitidinal fasciculus and inferior fronto occipital fasciculus)	34.1×-19.3×-6.8	JHU
VDSM	FDR (>0.05)	Z=1.88	
	Permutation FWE (>0.05)	Z=4.00	
	Tract (HCP 1065 atlas)		
	Corticothalamic pathway		
	Corpus Callosum		
	Thalamic radiata superior		

Significant Brain Lesion area in Hypoesthesia with Touch			
VLSM	FDR (>0.05)	Z=1.80	
	Permutation FWE (>0.05)	Z=3.60	
	Area	XYZ	Atlas
	Hippocampus	34.9×-27.4×-3.9	ALL
	Retrolenticular part of internal capsule	34.9×-27.4×-1.7	JHU
	Sagittal stratum (include inferior longitidinal fasciculus and inferior fronto occipital fasciculus)	34.9×-27.4×-5.3	JHU
VDSM	FDR (>0.05)	Z=2.05	
	Permutation FWE (>0.05)	Z=4.00	
	Tract (HCP 1065 atlas)		
	Inferior longitudinal fasciculus		

Significant Brain Lesion area in Hypoesthesia with VDT			
VLSM	FDR (>0.05)	Z=2.05	
	Permutation FWE (>0.05)	Z=3.424	
	Area	XYZ	Atlas
	External capsule	29.7×-1.6×-15.9	

	Superior longitudinal fasciculus	29.7×-2.3×-18.9	JHU
	FDR (>0.05)	Z=2.18	
	Permutation FWE (>0.05)	Z=3.62	
VDSM	Tract (HCP 1065 atlas)		
	Reticulospinal tract		
	Cingulum Frontal Parietal		

Each table presents the statistical ratio (Z ratio) and significantly detected areas in VLSM and VDSM. The volume of regions of VLSM presents x,y, and z. VLSM and VDSM used the JHU atlas, the AAL atlas, and the HCP 1065 atlas respectively. FDR, P<0.05, permutation family-wise error, P<0.05. FDR, false discovery rate; FWE, family-wise error; VLSM, voxel-based lesion-symptom mapping; VDSM, voxel-based disconnection-symptom mapping; JHU, the Johns Hopkins University white-matter tractography atlas; AAL, the Automated Anatomical Labelling atlas; HCP, Human Connectome Project atlas; VDT, vibration detection threshold; CPSP, central post-stroke pain; non-CPSP, non-central post-stroke pain.

Supplementary Fig 4

The results of VLSM in CPSP and non-CPSP groups

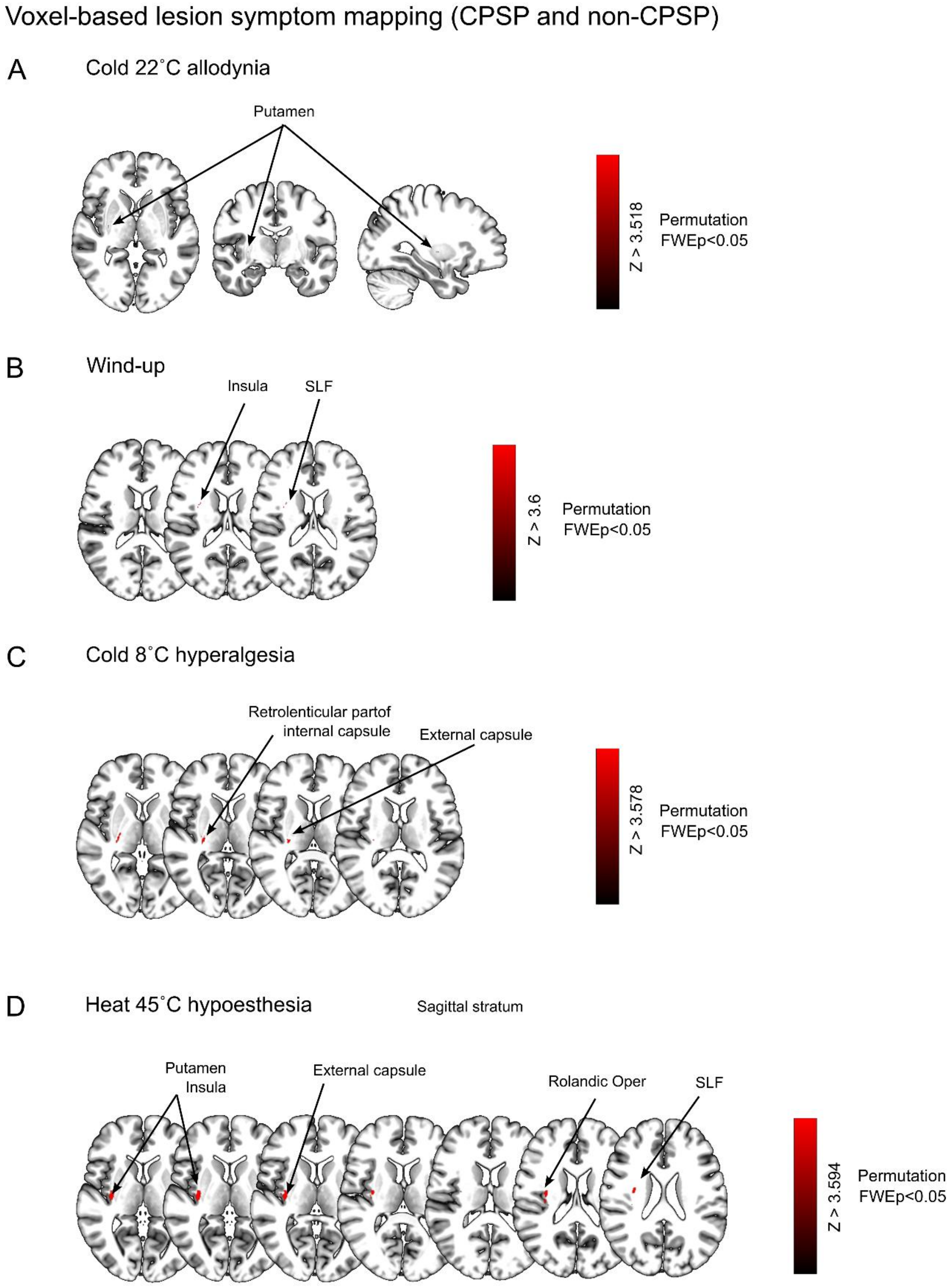

(**A**–**D**) The VLSM results indicate the areas associated with significant items in the bedside-QST among the no-pain and CPSP groups (only voxels involving at least 10 patients were included in VLSM). The color bars represent *Z*-scores; color-highlighted voxels are expressed at above-threshold regions (cold allodynia 22°C: $Z = 3.518$; WUR: $Z = 3.6$; cold hyperalgesia 8°C: $Z = 3.578$; heat hypoesthesia at 45°C: $Z = 3.594$). The lesion map in the VLSM presents only the right side (false-discovery rate, $P < 0.05$, permutation family-wise error, $P < 0.05$). CPSP, central post-stroke pain; non-CPSP, non-central post-stroke pain; WUR, wind-up; SLF, superior longitudinal fasciculus; FDR, false discovery rate; FWE, family-wise error; VLSM, voxel-based lesion–symptom mapping.

Supplementary Fig. 7

The results of VDSM in CPSP and non-CPSP groups

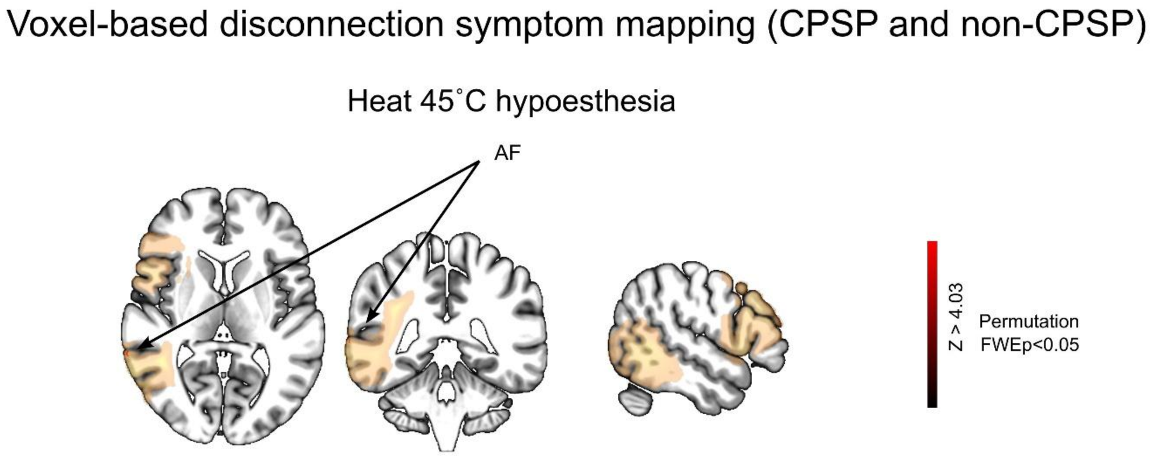

The VDSM results indicate the disconnection of white matter associated with heat hypoesthesia at 45°C in the bedside-QST among the non-CPSP and CPSP groups (20% of the sample). The color bars represent *Z*-scores; color-highlighted disconnections are shown at above-threshold regions (non-CPSP and CPSP groups: $Z > 4.03$). CPSP, central post-stroke pain; non-CPSP, non-central post-stroke pain; FDR, false discovery rate; FWE, family-wise error; AF, arcuate fasciculus; VDSM, voxel-based disconnection–symptom mapping.

Supplementary Table 7

Statistical ratio and brain areas in the CPSP and non-CPSP groups.

Significant Brain Lesion area in allodynia with Cold stimulation22˚C			
VLSM	FDR (>0.05)	Z=3.07	
	Permutation FWE (>0.05)	Z=3.501	
	Area	XYZ	Atlas
	Putamen	30.4×-10.4×0.5	ALL
VDSM	FDR (>0.05)	n.s	
	Permutation FWE (>0.05)	n.s	

Significant Brain Lesion area in hyperalgesia with Cold stimulation 8˚C			
VLSM	FDR (>0.05)	Z=2.08	
	Permutation FWE (>0.05)	Z=3.58	
	Area	XYZ	Atlas
	Retrolenticular part of internal capsule	27.4×-21.5×6.4	JHU
	External capsule	28.2×-21.5×9.3	JHU
VDSM	FDR (>0.05)	n.s	
	Permutation FWE (>0.05)	n.s	

Significant Brain Lesion area in Hypersensitivity with WUR			
VLSM	FDR (>0.05)	Z=3.26	
	Permutation FWE (>0.05)	Z=3.535	
	Area	XYZ	Atlas
	Superior longitudinal fasciculus	29.7×-2.1×18.2	JHU
	Insula	32.6×-1.6×17.4	JHU
VDSM	FDR (>0.05)	n.s	
	Permutation FWE (>0.05)	n.s	

Significant Brain Lesion area in Hypoesthesia with Heat stimulation 45˚C			
VLSM	FDR (>0.05)	Z=2.07	
	Permutation FWE (>0.05)	Z=3.59	
	Area	XYZ	Atlas
	Insula	34.9×-13.4×4.9	ALL
	Putamen	33.4×-14.9×4.7	ALL
	Rolandic Oper	34.8×-19.3×18.9	ALL

	Superior longitudinal fasciculus	31.9×-7.5×21.9	JHU
	External capsule	31.9×-12.6×6.4	JHU
VDSM	FDR (>0.05)	Z=1.99	
	Permutation FWE (>0.05)	Z=4.03	
	Tract (HCP 1065 atlas)		
	Arcuate fasciculus		

Each table presents the statistical ratio (Z ratio) and significantly detected areas in VLSM and VDSM. The volume of regions of VLSM presents x,y, and z. VLSM and VDSM used the JHU atlas, the AAL atlas, and the HCP 1065 atlas respectively. FDR, P<0.05, permutation family-wise error, P<0.05. FDR, false discovery rate; FWE, family-wise error; VLSM, voxel-based lesion-symptom mapping; VDSM, voxel-based disconnection-symptom mapping; JHU, the Johns Hopkins University white-matter tractography atlas; AAL, the Automated Anatomical Labelling atlas; HCP, Human Connectome Project atlas; WUR, wind-up ratio; CPSP, central post-stroke pain; non-CPSP, non-central post-stroke pain.

Supplementary Fig 5

The results of VLSM in no-pain and non-CPSP groups

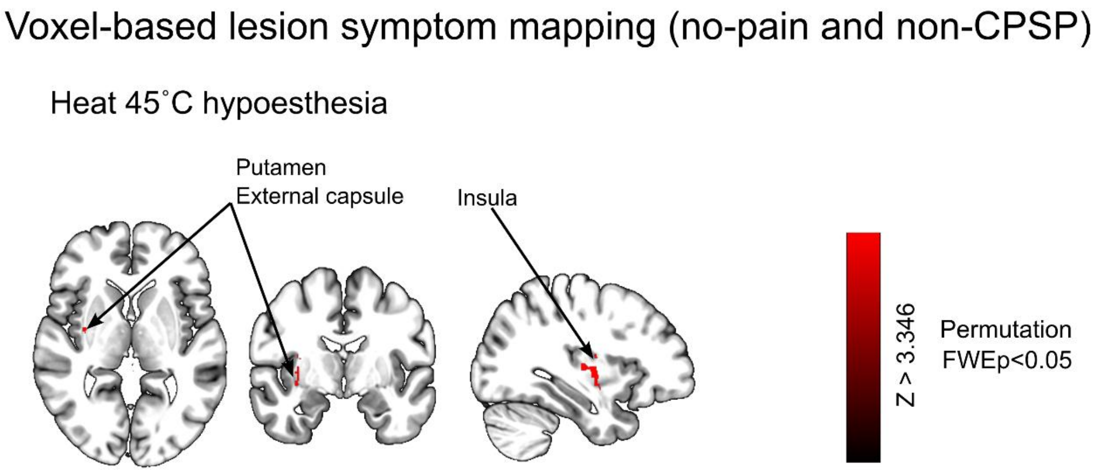

The VLSM results indicate the areas associated with heat hypoesthesia at 45°C in the bedside-QST among the no-pain and non-CPSP groups (only voxels involving at least 9 patients were included in VLSM). The color bars represent *Z*-scores; color-highlighted voxels are expressed at above-threshold regions (heat hypoesthesia at 45°C: $Z = 3.346$). The lesion map in the VLSM presents only the right side (false-discovery rate, $P < 0.05$, permutation family-wise error, $P < 0.05$). non-CPSP, non-central post-stroke pain; FDR, false discovery rate; FWE, family-wise error; VLSM, voxel-based lesion–symptom mapping.

Supplementary Fig 8

The results of VDSM in no-pain and non-CPSP groups

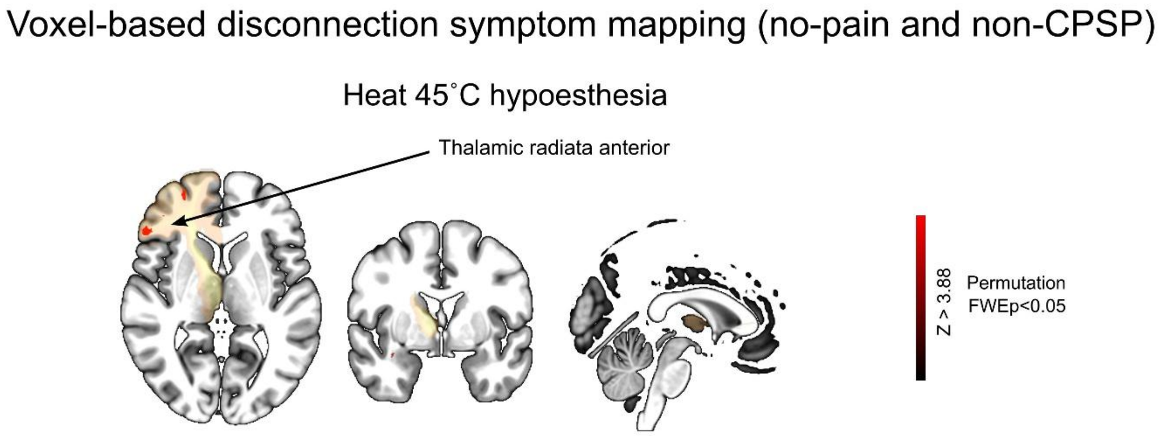

The VDSM results indicate the disconnection of white matter associated with heat hypoesthesia at 45°C in the bedside-QST among the non-CPSP and no-pain groups (20% of the sample). The color bars represent *Z*-scores; color-highlighted disconnections are shown at above-threshold regions (non-CPSP and no-pain groups: $Z > 3.88$). non-CPSP, non-central post-stroke pain; FDR, false discovery rate; FWE, family-wise error; VDSM, voxel-based disconnection–symptom mapping.

Supplementary Table 8

Statistical ratio and brain areas in the no-pain and non-CPSP groups

Significant Brain Lesion area in Hypoesthesia with Heat stimulation 45˚C			
VLSM	FDR (>0.05)	Z=2.02	
	Permutation FWE (>0.05)	Z=3.346	
	Area	XYZ	Atlas
	Insula	33.4×-6×13.8	ALL
	Putamen	34.1×-6.7×2	ALL
	External_capsule	33.3×-6.7×2	JHU
VDSM	FDR (>0.05)	Z=2.02	
	Permutation FWE (>0.05)	Z=3.88	
	Tract (HCP 1065 atlas)		
	Thalamic radiata anterior		

The table presents the statistical ratio (Z ratio) and significantly detected areas in VLSM and VDSM. The volume of regions of VLSM presents x,y, and z. VLSM and VDSM used the JHU atlas, the AAL atlas, and the HCP 1065 atlas respectively. FDR, P<0.05, permutation family-wise error, P<0.05. FDR, false discovery rate; FWE, family-wise error; VLSM, voxel-based lesion-symptom mapping; VDSM, voxel-based disconnection-symptom mapping; JHU, the Johns Hopkins University white-matter tractography atlas; AAL, the Automated Anatomical Labelling atlas; HCP, Human Connectome Project atlas; CPSP, central post-stroke pain; non-CPSP, non-central post-stroke pain
